# Efficient method for propargylation of aldehydes promoted by allenylboron compounds under microwave irradiation

**DOI:** 10.3762/bjoc.16.19

**Published:** 2020-02-04

**Authors:** Jucleiton J R Freitas, Queila P S B Freitas, Silvia R C P Andrade, Juliano C R Freitas, Roberta A Oliveira, Paulo H Menezes

**Affiliations:** 1 Universidade Federal Rural de Pernambuco - UACSA, PE, Brazil; 2 Departamento de Química Fundamental, Universidade Federal de Pernambuco, UFPE, Recife, Pernambuco 50740-560, Brazil; 3 Universidade Federal de Campina Grande, Centro de Educação e Saúde: Cuité, Paraíba, Brazil

**Keywords:** boron compounds, microwave, propargylation, regioselectivity, synthesis

## Abstract

The propargylation of aldehydes promoted by microwave irradiation using allenylboron compounds in a chemo- and regioselective way is described. The corresponding products were obtained in short reaction time, high yield and purity without the need of any solvent when allenylboronic acid pinacol ester was used, or using a minimal amount of acetone when potassium allenyltrifluoroborate was used.

## Introduction

The propargylation of carbonyl compounds is widely used in the synthesis of biologically active natural products [1]. Some examples can be found in the synthesis of histrionicotoxin [2], rhizopodin [3], baﬁlomycin [4], bryostatin [5], vancosamine [6] and macrolactin A [7].

Although there are several stereoselective methods described for the reaction of propargyl or allenyl organometallics with carbonyl compounds [8–14], the control of the regioselectivity is still a major concern. This is mainly due to the metallotropic rearrangement of propargyl and allenyl organometallics in solution resulting in mixtures of the two reagents [15]. Thus, upon reaction with an aldehyde, mixtures of propargylic and allenic alcohols can be obtained through a chelate transition state (S_E_2').

Attempts to improve the regioselectivity of the propargylation reaction by using allenic organometallic species of Cd [16], Ga [17], In [18], Ti [19], Al [20] and Bi [21] were described. However, the majority of these methods involve reagents that are difficult to prepare and to handle due to the sensitivity to air and moisture.

The use of less reactive species based on tin [22–24], silicon [25] or boron [26–28] to perform propargylation reactions typically requires catalysis by Lewis acids or bases and although the utility of allenylstannanes is further indicated by the commercial availability of some of them, the toxicity of these compounds makes them inappropriate for the use in pharmaceutical synthesis [29]. Moreover, the removal of tributyltin residues from reaction mixtures is also a major issue.

The use of microwave irradiation for the formation of new C–C bonds is nowadays widely used and offers several advantages such as the increment in the product yield, reduction of reaction time and the possibility to perform solvent-free reactions [30]. However, the relative “greenness” of microwave-assisted reactions is still a point of discussion. For example, the question about the energy efficiency of microwave vs conventionally heated reactions must, in general, be evaluated with great care on a case-by-case basis. Even so, the search for safer alternatives to current synthetic methodologies avoiding the use of moisture/air-sensitive organometallics and, more important, the development of solvent-free protocols are in accordance with the concept of environmental impact factor (*E* factor) [31].

Within this context, the development of solvent-free methods is highly desirable since the difficult for solvent recycling in academic laboratories and chemical manufacturing plants is universal. In addition, a reliable method for the propargylation reaction which could involve the use of commercially available and stable allenyl or propargyl compounds without the need of special conditions such as dry solvents or complex catalysts is a subject of great interest.

## Results and Discussion

For preliminary optimization of the reaction conditions, 2-naphthaldehyde (1 mmol) and allenylboronic acid pinacol ester (**1**, 1.5 mmol) in a capped vial were irradiated in a MW synthesizer for 30 minutes under different temperatures. The results are depicted in Table 1.

**Table 1:** Propargylation of 2-naphthaldehyde with allenylboronic acid pinacol ester (**1**) at different temperatures.^a^

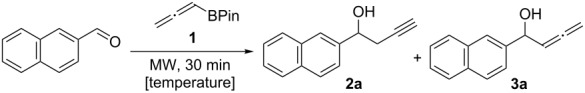			

Entry	Temp. (°C)	**2a**/**3a**^b^	Yield (%)^c^

1	75	97:3	94
2	100	98:2	97
3	125	98:2	81
4	150	98:2	84

^a^Reaction conditions: Reactions were performed with 2-naphthaldehyde (1 mmol), **1** (1.5 mmol) under MW irradiation (300 W) for 30 min at the temperature indicated. ^b^Determined by GC analysis. ^c^Isolated yield.

In all cases, the desired propargylic product **2a** was obtained together with a small amount of the corresponding allenic product **3a**. When the reaction was performed at 75 °C, good conversions of 2-naphthaldehyde into the corresponding products were observed (Table 1, entry 1). The best result was observed when the temperature was increased to 100 °C affording the products in 97% yield in a 98:2 ratio (Table 1, entry 2). Higher temperatures gave lower yields or the decomposition of the boron reagent **1** (Table 1, entries 3 and 4). It is worth to note that when the reaction was carried out with conventional heating (100 °C), the desired product was not observed after 1 h. A similar behavior was previously observed by Schaus [32].

Next, the shortest time necessary for the formation of the products at 100 °C under microwave irradiation was evaluated. The results are shown in Table 2. From Table 2, it can be seen that the increment in the reaction time resulted in higher yields without major changes in the ratio of **2a**:**3a**.

**Table 2:** Propargylation of 2-naphthaldehyde with allenylboronic acid pinacol ester (**1**) using different reaction times.^a^

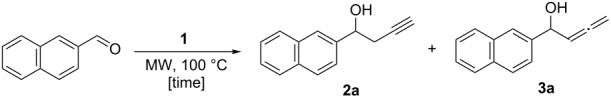			

Entry	Time (min)	**2a**/**3a**^b^	Yield (%)^c^

1	5	98:2	87
2	10	98:2	88
3	15	97:3	88
4	20	98:2	95
5	30	97:3	97

^a^Reaction conditions: Reactions were performed with 2-naphthaldehyde (1 mmol), **1** (1.5 mmol) under MW irradiation (300 W) at 100 °C for the time indicated. ^b^Determined by GC analysis. ^c^Isolated yield.

**Scheme 1:**
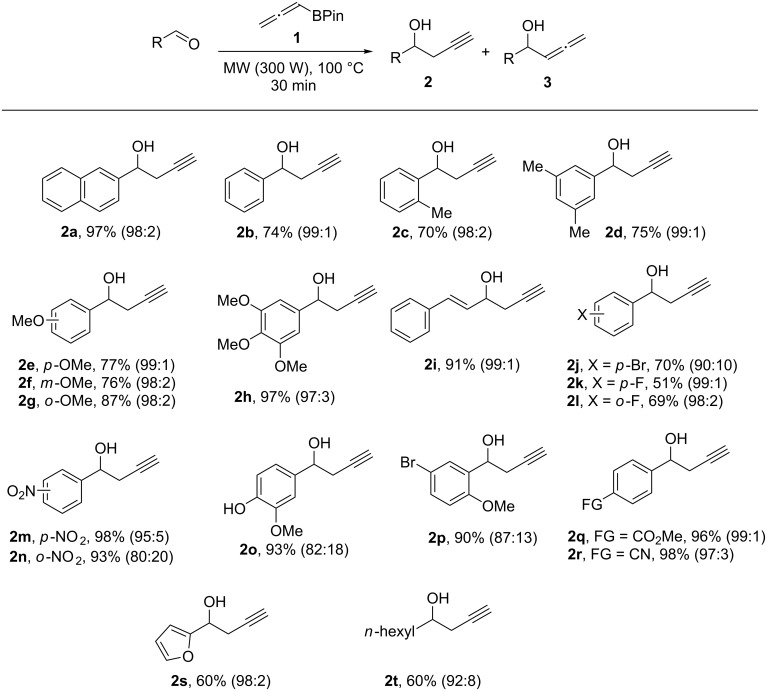
Scope of the propargylation reaction. Reactions were performed with the appropriate aldehyde (1 mmol), **1** (1.5 mmol) under microwave irradiation (300 W) at 100 °C for 30 min. Isolated yields are given. The number in parentheses refers to the mixture of propagyl and allenyl regioisomers determined by GC analysis.

The optimized reaction conditions, namely: 2-naphthaldehyde (1.0 mmol), **1** (1.5 mmol) under microwave irradiation (300 W) were then applied for the propargylation reaction of aldehydes containing a wide range of functional groups and the results are shown in Scheme 1. In all cases the reaction proceeded smoothly leading to the conversion of aldehydes into the corresponding homopropargylic alcohols **2** in moderate to high yields and in a very regioselective way, while the propargylated product was obtained as the major product in all cases.

Aromatic aldehydes such as benzaldehyde, 2-methylbenzaldehyde and 3,5-dimethylbenzaldehyde gave the corresponding products **2b**–**d** in good yields in a regioselective way regardless of the position of the substituent on the aromatic ring.

When aldehydes containing electron-donating groups such as 2-, 3- or 4-methoxybenzaldehyde or 3,4,5-trimethoxybenzaldehyde were used, the corresponding products **2e**–**h** were obtained in good yields and regioselectivities. In the same way, aldehydes containing electron-withdrawing groups such as the nitro group also reacted without influence of the substituent location to give the corresponding homopropargylic alcohols **2m** and **2n** in good yields. It is interesting to note that the nitro group remained intact under the reaction conditions. Usually, this group is sensitive to reduction when alternative methods involving zinc or tin are used [33].

In addition, aldehydes containing halogens also gave the corresponding products **2j**–**l** in moderate yields. These results indicated that the substituent nature, whether electron-donating or electron-withdrawing, has no dramatic inﬂuence on the product yields.

When the α,β-unsaturated aldehyde, cinnamaldehyde was used, the corresponding 1,2-addition product **2i** was obtained exclusively.

The chemoselectivity of the method was evaluated using aldehydes containing different functionalities. For example, the use of vanillin, an aldehyde containing the acidic phenol group as substituent, gave the corresponding product **2o** in 93% yield in an 82:18 ratio of regioisomers. In the same way, when aldehydes containing an ester or nitrile group were used, the corresponding products **2q** and **2r** were obtained in good yields. The use of a heteroaromatic or an aliphatic aldehyde as substrates under the optimized conditions gave the corresponding homopropargylic alcohols **2s**,**t** in moderate yields.

The development of a solvent-free protocol for propargylation of aldehydes based on microwave irradiation should take into account not only the reaction itself but also an effective method for the extraction of the obtained products. Despite the excellent results described on Scheme 1, a factor that must be taken into consideration is the removal of the desired alcohols **2a**–**t** from pinacol – the byproduct obtained in the reaction. There are some examples in the literature based on the removal of pinacol by distillation in vacuo (50 °C/0.05 mbar) [34]. However, this technique can only be applied for non-volatile samples. More recently, Aggarwal and co-workers [35] described an efficient method for removal of pinacol based on the formation of an azeotrope with water under moderate vacuum. Thus, a test experiment was performed using compound **2h**. The crude product obtained from the reaction was dissolved in 50% aqueous methanol and the volatile materials were removed using a rotary evaporator. The procedure was monitored by gas chromatography and was repeated until the crude mixture contained less than 1 mol % of pinacol remaining. The results are described in Table 3.

**Table 3:** Number of cycles to remove pinacol from the crude product.^a^

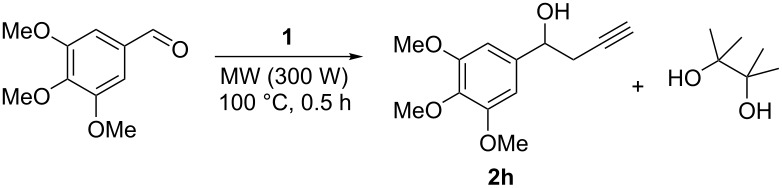		

Entry	Cycle	Ratio **2h**/pinacol (%)^b^

1	1	70:30
2	2	77:23
3	3	84:16
4	4	93:7
5	5	99:1

^a^Reaction conditions: The reaction was performed using 3,4,5-trimethoxybenzaldehyde (1 mmol) and **1** (1.5 mmol) under microwave irradiation (300 W) at 100 °C for 0.5 h. The crude product was dissolved in 50% aqueous methanol (10 mL) and the solvents were removed on a rotary evaporator (45–50 °C/25–15 mbar). ^b^Determined by GC analysis.

**Scheme 2:**
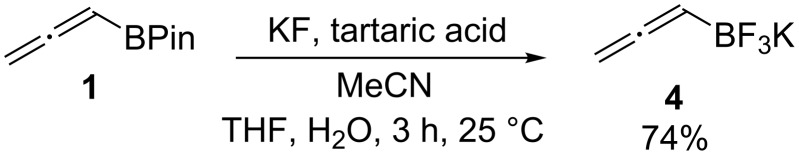
Synthesis of potassium allenyltrifluoroborate (**4**).

Although the desired products **2a**–**t** having been obtained along with a small proportion of the corresponding regioisomer in some cases, in the development of new synthetic methods, it is desirable that it gives the corresponding product as a single compound. Our group described the synthesis of homopropargylic alcohols using potassium allenyltrifluoroborate as the propargylating agent in a very regioselective way [36–37]. Thus, allenylboronic acid pinacol ester (**1**) was converted into the corresponding trifluoroborate using the procedure described by Lloyd-Jones and co-worker [38]. The desired product **4** was obtained in good yield and characterized by ^1^H, ^13^C, ^11^B and ^19^F NMR [39] (Scheme 2).

Potassium allenyltrifluoroborate (**4**) is a crystalline solid and despite several microwave promoted reactions can be conducted without the use of solvents, the propargylation reaction using 2-naphthaldehyde and **4** under the previously optimized conditions gave the desired product in only low conversion (Table 4, entry 1).

**Table 4:** Propargylation of 2-naphthaldehyde with potassium allenyltrifluoroborate (**4**) using different solvents.^a^

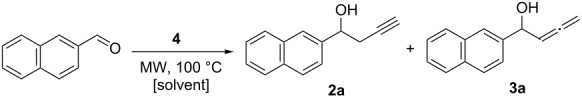				

Entry	Solvent	Time (min)	**2a**/**3a**^b^	Yield (%)^c^

1	–	–	–	6
2	acetone	30	100:0	92
3	water	30	100:0	3
4	acetone/water	30	100:0	10
5	ethylene glycol	20	100:0	45
6	ethanol	20	100:0	45
7	methanol	20	100:0	30
8	dichloromethane	20	100:0	10

^a^Reaction conditions: Reactions were performed with 2-naphthaldehyde (1 mmol), **4** (1.5 mmol) under MW irradiation (300 W) at 100 °C for the time indicated using the appropriate solvent (500 μL). ^b^Determined by GC analysis. ^c^Isolated yield.

In order to find an appropriate and suitable solvent that could be used in small amounts to promote the reaction different solvents were screened and the best result was observed when a small amount of acetone was used, where **2a** was obtained in 92% yield as a single regioisomer (Table 4, entry 2). Low conversions were observed when water or a 1:1 mixture of acetone and water was used in the reaction (Table 4, entries 3 and 4). The use of alcohols also gave the desired product in lower yields (Table 4, entries 5–7). Finally, when a less polar solvent was used, the observed conversion was only 10% (Table 4, entry 8).

Two factors must be considered when allenylboron species are used in the propargylation reaction. The first is that the atomic efficiency [40] using potassium allenyltrifluoroborate (**4**, 62%) or allenylboronic acid pinacol ester (**1**, 58%) is higher when compared to the commercially available allenyltributyltin (22%) for similar reactions.

**Scheme 3:**
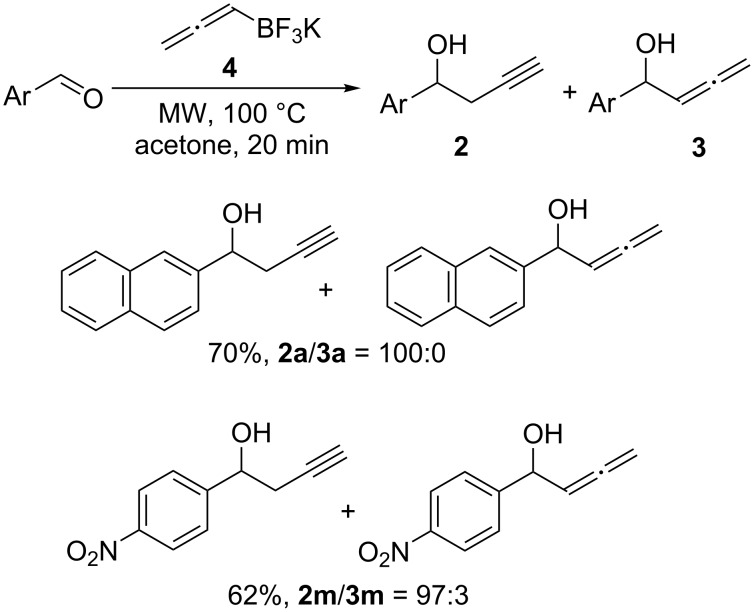
Propargylation of aldehydes using potassium allenyltrifluoroborate (**4**).

The second refers to the regioselectivity of the reaction. Despite the reaction using 2-naphthaldehyde led only to the propargyl isomer **2a**, when the same reaction conditions were applied to 4-nitrobenzaldehyde, the desired product **2m** was once again obtained with a small amount of the corresponding regioisomer **3m** in a 97:3 ratio (Scheme 3).

## Conclusion

In summary, we have shown an efficient method for the propargylation of aldehydes promoted by microwave irradiation using allenylboron compounds in a chemo- and regioselective way. The corresponding products were obtained after a short reaction time in high yield and purity without the need of a solvent when allenylboronic acid pinacol ester was used, or using a minimal amount of acetone when potassium allenyltrifluoroborate was used. The method is simple, fast and general allowing further applications in the synthesis of more complex compounds.

## Supporting Information

File 1 Experimental procedures and ^1^H, ^13^C, ^11^B and ^19^F NMR spectra for all synthesized compounds.
