# Supplementary material for: Efficient method for propargylation of aldehydes promoted by allenylboron compounds under microwave irradiation
Source: Beilstein J Org Chem. 2020 Feb 4;16:168–74. doi: 10.3762/bjoc.16.19 (PMC7034246; doi:10.3762/bjoc.16.19)
Supplement: File 1 — Experimental procedures and 1H, 13C, 11B and 19F NMR spectra for all synthesized compounds. [file Beilstein_J_Org_Chem-16-168-s001.pdf]

BEILSTEIN JOURNAL OF ORGANIC CHEMISTRY

Supporting Information

for

Efficient method for propargylation of aldehydes promoted by allenylboron compounds under microwave irradiation

Jucleiton J. R. Freitas, Queila P. S. B. Freitas, Silvia R. C. P. Andrade,
Juliano C. R. Freitas, Roberta A. Oliveira and Paulo H. Menezes

Beilstein J. Org. Chem. **2020,** *16,* 168–174. doi:10.3762/bjoc.16.19

Experimental procedures and ^{1}H, ^{13}C, ^{11}B and ^{19}F NMR spectra for all synthesized compounds

License and Terms: This is a supporting information file under the terms of the Creative Commons Attribution License (https://creativecommons.org/licenses/by/4.0). Please note that the reuse, redistribution and reproduction in particular requires that the authors and source are credited.
The license is subject to the *Beilstein Journal of Organic Chemistry* terms and conditions: (https://www.beilstein-journals.org/bjoc)

1. General……… S2
2. Experimental procedures.. S2
3. Spectra.. S7

1. General

1.1 Materials and methods

All reagents and solvents used were previously purified and dried in agreement with the literature [1]. The aldehydes and allenylboronic acid pinacol ester, **1** were purchased from Aldrich Chemical Co. and used as received. Reactions were monitored by thin-layer chromatography on 0.25 mm E. Merck silica gel 60 plates (F_{254}) using UV light, vanillin and *p*-anisaldehyde as visualizing agents. The reactions were performed using a microwave reactor CEM Focused Microwave Synthesis Discover System Model 908005 using a 10 mL IntelliVent reactor. The GC/FID analysis was performed on a Varian CP-3380 gas chromatograph system coupled with a flame ionization detector. The chromatographic column was a Chrompack CP-SPL5CB capillary column (30 m × 0.25 mm, 0.25 µm) using the initial temperature 60 °C then increased to 220 °C at 10 °C min^{-1}.^{1}H and ^{13}C NMR data were recorded in $CDCl_3$ or DMSO-d_6. The chemical shifts are reported as delta (δ) units in parts per million (ppm) relative to the solvent residual peak as the internal reference [$CDCl_3$ at δ 7.26 (^{1}H NMR) and δ 77.16 (^{13}C NMR) and DMSO-d_6 at δ 2.50 (^{1}H NMR) and δ 39.52 (^{13}C NMR)]. ^{11}B (128 MHz) and ^{19}F NMR (376 MHz) spectra were obtained in DMSO-d_6. Spectra were calibrated using BF_3•Et_2O (0.0 ppm) as external reference in the case of ^{11}B NMR and chemical shifts were referenced to external CF_3CO_2H (0.0 ppm) in the case of ^{19}F NMR. Coupling constants (*J*) for all spectra are reported in Hertz (Hz).

2. Experimental procedures

2.1 General procedure for the propargylation of aldehydes using allenylboronic acid pinacol ester (1) promoted by microwave irradiation: A vial containing the appropriate aldehyde (1 mmol) and allenylboronic acid pinacol ester, **1** (1.5 mmol, 250 mg) was irradiated at 300 W at 100 °C for 30 minutes. The vial contents were diluted with EtOAc (10 mL) and washed with water (2 × 15 mL). The combined organic phase was dried over $MgSO_4$, filtered, and the solvent was removed in vacuo followed by purification by a flash column chromatography [hexanes/EtOAc (8:2)] to yield **2a**–**t**.

1-(Naphth-2-yl)but-3-yn-1-ol (**2a**)*:* obtained 190 mg (97%) as a pale yellow oil. ^{1}H NMR (400 MHz, $CDCl_3$) δ 7.79-7.75 (m, 4H, $H_{naphthyl}$), 7.44–7.39 (m, 2H, $H_{naphthyl}$), 7.18 (s, 1H, $H_{naphthyl}$), 4.98 (t, *J* = 6.4 Hz, 1H, OC*H*CH_2), 2.68–2.65 (m, 2H, OCHC*H_2*), 2.01 (t, *J* = 2.8 Hz, 1H, C≡CH); ^{13}C NMR (100 MHz, $CDCl_3$) δ 139.8, 133.2, 133.1, 128.3, 128.0, 127.7, 126.2, 126.0, 124.6, 123.7, 80.6, 72.4, 71.1, 29.4. The data match with the previously described compound [2].

1-Phenyl-3-butyn-1-ol (**2b**)*:* obtained 108 mg (74%) as an oil. ^{1}H NMR (400 MHz, $CDCl_3$) δ 7.42–7.32 (m, 5H, H_{aryl}), 4.90 (t, *J* = 6.3 Hz, 1H, OC*H*CH_2), 2.67–2.65 (m, 2H, OCHC*H_2*), 2.10 (t, *J* = 2.8 Hz, 1H, C≡CH); ^{13}C NMR (100 MHz, $CDCl_3$) δ 142.5, 128.4, 127.9, 125.7, 80.7, 72.2, 70.9, 29.4. The data match with the previously described compound [2].

1-(o-Tolyl)but-3-yn-1-ol (**2c**)*:* obtained 112 mg (70%) as an oil. ^{1}H NMR (300 MHz, $CDCl_3$) δ 7.53–7.50 (m, 1H, H_{aryl}), 7.28–7.13 (m, 3H, H_{aryl}), 5.12 (dd, *J* = 6.0 and 5.7 Hz, 1H, OC*H*CH_2), 2.69–2.54 (m, 2H, OCHCH_2), 2.37 (s, 3H, CH_3), 2.12 (br s, 1H, O*H*), 2.09 (t, *J* = 2.4 Hz, 1H, C≡CH); ^{13}C NMR (75 MHZ, $CDCl_3$) δ 140.4, 134.6, 130.4, 127.7, 126.3, 125.0, 80.9, 70.7, 68.8, 28.2, 19.0. The data match with the previously described compound [3].

1-(2,5-Dimethylphenyl)but-3-yn-1-ol (**2d**)*:* obtained 130 mg (75%) as an oil.^{1}H NMR (400 MHz, $CDCl_3$) δ 7.34 (s, 1H, H_{aryl}), 7.06–7.01 (m, 2H, H_{aryl}), 5.12–5.08 (m, 1H, OC*H*CH_2), 2.66–2.55 (m, 2H, OCHCH_2), 2.34 (s, 3H, CH_3), 2.33 (s, 3H, CH_3), 2.10 (t, *J* = 2.8 Hz, 1H, C≡CH). ^{13}C NMR (100 MHz, $CDCl_3$) δ 140.2, 135.8, 131.4, 130.4, 128.4, 125.6, 80.0, 70.6, 68.9, 28.3, 21.1, 18.6. The data match with the previously described compound [4].

1-(p-Methoxyphenyl)but-3-yn-1-ol (**2e**)*:* obtained 135 mg (77%) as an oil.^{1}H NMR (400 MHz, $CDCl_3$) δ 7.33 (d, *J* = 8.0 Hz, 2H, H_{aryl}), 6.91 (d, *J* = 8.8 Hz, 2H, H_{aryl}), 4.85 (t, *J* = 6.4 Hz, 1H, OC*H*CH_2), 3.82 (s, 3H, OCH_3), 2.65–2.63 (m, 2H, OCHCH_2), 2.08 (t, *J* = 2.4 Hz, 1H, C≡CH); ^{13}C NMR (100 MHz, $CDCl_3$) δ 159.3, 134.6, 127.0, 113.9, 80.8, 72.0, 70.9, 55.3, 29.4. The data match with the previously described compound [2].

1-(m-Methoxyphenyl)but-3-yn-1-ol (**2f**)*:* obtained 133 mg (76%) as an oil. ^{1}H NMR (400 MHz, $CDCl_3$) δ 7.23–7.18 (m, 1H, H_{aryl}), 6.90–6.88 (m, 2H, H_{aryl}), 6.79–6.76 (m, 1H, H_{aryl}), 4.78 (t, *J* = 6.4 Hz, 1H, OC*H*CH_2), 3.75 (s, 3H, OCH_3), 2.58–2.56 (m, 2H, OCHCH_2), 2.00 (t, *J* = 2.8 Hz, 1H, C≡CH); ^{13}C NMR (100 MHz, $CDCl_3$) δ 159.7, 144.1, 129.5, 118.0, 113.4, 111.2, 80.6, 72.2, 70.9, 55.2, 29.4. The data match with the previously described compound [2].

1-(o-Methoxyphenyl)but-3-yn-1-ol (**2g**)*:* obtained 153 mg (87%) as an oil. ^{1}H NMR (400 MHz, $CDCl_3$) δ 7.42 (dd, *J* = 7.6, 1.6 Hz, 1H, H_{aryl}), 7.31–7.26 (m, 1H, H_{aryl}), 6.99 (dt, *J* = 7.6, 0.8 Hz, 1H, H_{aryl}), 6.90 (d, *J* = 8.4 Hz, 1H, H_{aryl}), 5.10 (dd, *J* = 7.6, 5.2 Hz, 1H, OC*H*CH_2), 3.87 (s, 3H, OCH_3), 2.78 (ddd, *J* = 16.8, 5.2, 2.8 Hz, 1H, OCHCH_2), 2.65 (ddd, *J* = 16.8, 7.2, 2.8 Hz, 1H, OCHCH_2), 2.06 (t, *J* = 2.8 Hz, 1H, C≡CH). ^{13}C NMR (100 MHz, $CDCl_3$) δ 156.2, 130.2, 128.7, 126.8, 120.7, 110.4, 81.3, 70.4, 68.9, 55.2, 27.4. The data match with the previously described compound [3].

1-(3,4,5-Trimethoxyphenyl)but-3-yn-1-ol (**2h**)*:* obtained 229 mg (97%) as an oil.^{1}H NMR (400 MHz, $CDCl_3$) δ 6.64 (s, 2H, H_{aryl}), 4.83 (dt, *J* = 6.4 and 2.8 Hz, 1H, OC*H*CH_2), 3.88 (s, 6H, OCH_3), 3.85 (s, 3H, OCH_3), 2.68–2.59 (m, 2H, OCHCH_2), 2.41 (d, *J* = 3.2 Hz, 1H, OH), 2.11 (t, *J* = 2.8 Hz, 1H, C≡CH). ^{13}C NMR (100 MHz, $CDCl_3$) δ 153.4, 138.2, 137.6, 102.7, 80.7, 72.5, 71.1, 60.8, 56.1, 29.6. The data match with the previously described compound [5].

(E)-1-Phenylhex-1-en-5-yn-3-ol (**2i**)*:* obtained 156 mg (91%) as an oil.^{1}H NMR (400 MHz, $CDCl_3$) δ 7.41 (d, *J* = 7.6 Hz, 2H, H_{aryl}), 7.34 (t, *J* = 8.0 Hz, 1H, H_{aryl}), 7.28–7.24 (m, 2H, H_{aryl}), 6.68 (d, *J* = 16.0 Hz, 1H, C*H*=CH), 6.30 (dd, *J* = 16.0 6.0 Hz, 1H, CH=C*H*), 4.52–4.47 (m, 1H, OC*H*CH_2), 2.61 (ddd, *J* = 16.8, 5.6 2.8 Hz, 1H, OCHCH_2), 2.54 (ddd, *J*= 16.8, 6.0 and 2.4 Hz, 1H, OCHCH_2),

2.10 (t, J = 2.4 Hz, 1H, C≡CH); ^{13}C NMR (100 MHz, $CDCl_3$) δ 136.3, 131.4, 129.9, 128.6, 127.9, 126.6, 80.2, 71.1, 70.7, 27.7. The data match with the previously described compound.[2]

1-(p-Bromophenyl)but-3-yn-1-ol (**2j**)*:* obtained 157 mg (70%) as an oil.^{1}H NMR (400 MHz, $CDCl_3$) δ 7.50 (d, J = 8.4 Hz, 2H, H_{aryl}), 7.29 (d, J = 8.4 Hz, 2H, H_{aryl}), 4.86 (t, J = 6.4 Hz, 1H, OC*H*CH_2), 2.68–2.57 (m, 2H, OCHC*H_2*), 2.09 (t, J = 2.8 Hz, 1H, C≡CH); ^{13}C NMR (100 MHz, $CDCl_3$) δ 141.3, 131.6, 127.5, 121.8, 80.1, 71.6, 71.3, 29.4. The data match with the previously described compound [3].

1-(p-Fluorophenyl)but-3-yn-1-ol (**2k**)*:* obtained 84 mg (51%) as an oil. ^{1}H NMR (400 MHz, $CDCl_3$) δ 7.40–7.36 (m, 2H, H_{aryl}), 7.08-7.04 (m, 2H, H_{aryl}), 4.88 (t, J = 6.4 Hz, 1H, OC*H*CH_2), 2.65-2.62 (m, 2H, OCHC*H_2*), 2.09 (t, J = 2.8 Hz, 1H, C≡CH); ^{13}C NMR (100 MHz, $CDCl_3$) δ 162.4 (d, J = 244.7 Hz), 138.1 (d, J = 3.1 Hz), 127.4 (d, J = 8.5 Hz), 115.3 (d, J = 21.7 Hz), 80.3, 71.7, 71.2, 29.6. The data match with the previously described compound [2].

1-(o-Fluorophenyl)but-3-yn-1-ol (**2l**)*:* obtained 113 mg (69%) as an oil. ^{1}H NMR (400 MHz, $CDCl_3$) δ 7.47 (t, J = 7.6 Hz, 1H, Ar), 7.22–7.19 (m, 1H, Ar), 7.10 (t, J = 7.6 Hz, 1H, Ar), 6.97 (t, J = 9.2 Hz, 1H, Ar), 5.13 (br, 1H, OC*H* CH_2), 2.71–2.67 (m, 1H, OCHC*H_2*), 2.59–2.54 (m, 1H, OCHC*H_2*), 2.10–1.91 (m, 1H, ≡CH). ^{13}C NMR (100 MHz, $CDCl_3$) δ 159.6 (d, J = 244.8 Hz), 129.3 (d, J = 8.0 Hz), 127.2, 124.3, 115.3 (d, J = 22.0 Hz), 80.2, 71.1, 66.4, 28.3. The data match with the previously described compound [3].

1-(p-Nitrophenyl)but-3-yn-1-ol (**2m**)*:* obtained 187 mg (98%) as an oil. ^{1}H NMR (400 MHz, $CDCl_3$) δ 8.23 (d, J= 8.8 Hz, 2H, H_{aryl}), 7.58 (d, J = 8.8 Hz, 2H, H_{aryl}), 4.99 (dd, 1H, J = 6.8 and 5.6 Hz, 1H, OC*H*CH_2), 2.70 (ddd, J = 16.8, 5.62.4 Hz, 1H, OCHC*H_2*), 2.63 (ddd, J = 16.8, 6.82.8 Hz, 1H, OCHC*H_2*), 2.11 (dd, J = 2.8 and 2.4 Hz, 1H, C≡CH); ^{13}C NMR (100 MHz, $CDCl_3$) δ 149.4, 147.5, 126.6, 123.7, 79.3, 72.0, 71.3, 29.5. The data match with the previously described compound [2].

1-(o-Nitrophenyl)but-3-yn-1-ol (**2n**)*:* obtained 177 mg (93%) as an oil. ^{1}H NMR (300 MHz, $CDCl_3$) δ 7.97 (dd, J = 8.1, 1.2 Hz, 1H, H_{aryl}), 7.90 (dd, J = 8.4 and 1.2 Hz, 1H, H_{aryl}), 7.69 (dt, J = 7.8 1.2 Hz, 1H, H_{aryl}), 7.48 (dt, J = 7.2, 1.2 Hz, 1H, H_{aryl}), 5.48 (dd, J = 7.2, 4.8 Hz, 1H, OC*H*CH_2), 2.92 (ddd, J = 16.5, 4.8, 3.0 Hz, 1H, OCHC*H_2*), 2.68 (ddd, J = 3.0, 7.5 and 16.5 Hz, 1H, OCHC*H_2*), 2.12 (t, J = 3.0 Hz, 1H, ≡CH). ^{13}C NMR (75 MHz, $CDCl_3$) δ 147.6, 137.7, 133.6, 128.6, 128.2, 124.5, 79.7, 71.8, 67.4, 28.5. The data match with the previously described compound [3].

4-(1-Hydroxybut-3-ynyl)-2-methoxyphenol (**2o**)*:* obtained 178 mg (93%) as an oil. ^{1}H NMR (400 MHz, $CDCl_3$) δ 6.95 (d, J = 2.0 Hz, 1H, H_{aryl}), 6.88 (d, J = 8.2 Hz, H_{aryl}), 6.85 (dd, J = 8.2, 1.6 Hz, 1H, H_{aryl}), 5.67 (br s, 1H, PhO*H*), 4.81 (t, J = 6.4 Hz, 1H, OC*H*CH_2), 3.89 (s, 3H, C*H_3*), 2.68–2.59 (m, 2H, OCHC*H_2*), 2.44 (br s, 1H, CHO*H*), 2.08 (t, J = 2.8 Hz, 1H, ≡CH). ^{13}C NMR (100 MHz, $CDCl_3$) δ 146.5, 145.3, 134.5, 118.8, 114.1, 108.2, 80.8, 72.2, 70.9, 55.9, 29.4. The data match with the previously described compound [6].

1-(5-Bromo-2-methoxyphenyl)but-3-yn-1-ol (**2p**)*:* obtained 228 mg (90%) as an oil. ^{1}H NMR (400 MHz, $CDCl_3$) δ 7.56 (d, J = 2.4 Hz, 1H, H_{aryl}), 7.36 (dd, J = 8.4 and 2.4 Hz, 1H, H_{aryl}), 6.75 (d, J =

8.8 Hz, 1H, H_{aryl}), 5.06 (dd, *J* = 7.6 and 4.8 Hz, 1 H, OC*H*CH_2), 3.83 (s, 3 H, CH_3), 2.75 (ddd, *J* = 16.8, 7.2 and 2.8 Hz, 1H, OCHCH_2), 2.56 (ddd, *J* = 16.8, 7.6 and 2.4 Hz, 1H, OCHCH_2), 2.08 (t, *J* = 2.8 Hz, 1H, C≡C*H*). ^{13}C NMR (100 MHz, $CDCl_3$) δ 155.1, 132.5, 131.2, 129.6, 113.1, 112.0, 80.7, 70.9, 67.7, 55.5, 27.4. The data match with the previously described compound [7].

Methyl 4-(1-Hydroxybut-3-ynyl)benzoate (**2q**)*:* obtained 195 mg (96%) as an oil. ^{1}H NMR (400 MHz, $CDCl_3$) δ 8.02 (d, *J* = 8.4 Hz, 2H, H_{aryl}), 7.46 (d, *J* = 8.0 Hz, 2H, H_{aryl}), 4.95–4.92 (m, 1H, OC*H*CH_2), 3.91 (s, 3H, CH_3), 2.71–2.59 (m, 2H, OCHCH_2), 2.59 (d, *J* = 3.6 Hz, 1H, OH), 2.08 (t, *J* = 2.8 Hz, 1H, C≡CH). ^{13}C NMR (100 MHz, $CDCl_3$) δ 166.8, 147.4, 129.7, 129.7, 125.7, 80.0, 71.8, 71.4, 52.1, 29.4. The data match with the previously described compound [8].

4-(1-Hydroxybut-3-yn-yl)benzonitrile (**2r**)*:* obtained 167 mg (98%) as an oil. ^{1}H NMR (400 MHz, $CDCl_3$) δ 7.67 (d, *J* = 8.0 Hz, 2H, H_{aryl}), 7.52 (d, *J* = 8.0 Hz, 2H, H_{aryl}), 4.95 (dd, *J* = 7.8 and 1.8 Hz, 1H, OC*H*CH_2), 2.73–2.57 (m, 2H, OCHCH_2), 2.11 (t, *J* = 2.8 Hz, 1H, C≡CH). ^{13}C NMR (100 MHz, $CDCl_3$) δ 147.5, 132.3, 126.5, 118.7, 111.8, 79.4, 71.9, 71.4, 29.4. The data match with the previously described compound [8].

1-(2-Furyl)but-3-yn-1-ol (**2s**)*:* obtained 82 mg (60%) as an oil. ^{1}H NMR (300 MHz, $CDCl_3$) δ 7.39 (t, *J* = 1.2 Hz, 1H H_{furyl}), 6.34 (d, 2H, *J* = 1.2 Hz, 1H, H_{furyl}), 4.88 (t, *J* = 6.0 Hz, 1H, OC*H*CH_2), 2.77 (dd, *J* = 6.0, 2.4 Hz, 2H, OCHCH_2), 2.33 (s, 1H, OH), 2.07 (t, *J* = 2.4 Hz, 1H, C≡CH); ^{13}C NMR (75 MHz, $CDCl_3$) δ 154.6, 142.3, 110.3, 106.6, 79.8, 71.2, 66.1, 26.1. The data match with the previously described compound [2].

Dec-1-yn-4-ol (**2t**)*:* obtained 92 mg (60%) as an oil. ^{1}H NMR (300 MHz, $CDCl_3$) δ 3.80–3.72 (m, 1H, OC*H*CH_2), 2.44 (ddd, *J* = 16.5, 4.8, 3.0 Hz, 1H, OCHCH_2), 2.31 (ddd, *J* = 16.5, 6.3, 3.0 Hz, 1H, OCHCH_2), 2.06 (t, *J* = 3,0 Hz, 1H, C≡CH), 1.56–1.51 (m, 2H, CH_2), 1.36–1.29 (m, 8H, (CH_2)$_4$), 0.88 (t, *J* = 6.3 Hz, 3H, CH_3); ^{13}C NMR (75 MHz, $CDCl_3$) δ 80.9, 70.7, 69.9, 36.2, 31.7, 29.2, 27.3, 25.5, 22.6, 14.0. The data match with the previously described compound.[9]

2.2 Representative procedure for propargylation of 2-naphthaldehyde using potassium allenyltrifluoroborate (4) promoted by microwave irradiation: A vial containing 2-naphthaldehyde (1 mmol, 156 mg) and potassium allenyltrifluoroborate (**4**, 1.5 mmol, 250 mg) in acetone (500 μL) was irradiated at 300 W at 100 °C for 20 minutes. The vial contents were diluted with EtOAc (10 mL) and washed with water (2 × 15 mL). The organic phase was dried over $MgSO_4$ and filtered. The solvents were removed in vacuo to yield **2a** (137 mg, 70%) as a single isomer.

2.3 Synthesis of potassium allenyltrifluoroborate (4): A solution of KF (4 equiv, 11 mmol, 0.64 g) in H_2O (1.0 mL) was added to a suspension of the allenylboronic acid pinacol ester (**1**, 0.45 g, 2.75 mmol) in MeCN/MeOH (1:1) (3.0 mL). The mixture was stirred until complete dissolution (aprox. 1 min). *L*-(+)-tartaric acid [2.05 equiv, 5.64 mmol, 0.84 g in THF (4.0 mL)] was added dropwise to the rapidly stirring biphasic solution over a period of approximately 5 min, during which a white precipitate formed and rapidly flocculated. After 4.0 h stirring, the mixture was filtered, washed with more MeCN and filtered. The filtrate was concentrated in vacuo to give **4** as

a white solid (0.29 g, 74%) [10]. ^{1}H NMR (400 MHz, DMSO-d_6): δ 4.51 (br s, 1H, C*H*BF$_3$K), 3.98 (br s, 2H, CH_2=C); ^{13}C NMR (100 MHz, DMSO-d_6): δ 210.0, 65.8; ^{11}B NMR (128 MHz, DMSO-d_6): δ 0.59 (q, $J_{11B,19F}$ = 49.2 Hz, BF$_3$K); ^{19}F NMR (376 MHz, DMSO-d_6): δ -133.75 (q, $J_{19F,11B}$ = 49.2 Hz, BF$_3$K). The data match with the previously described compound [11].

2.4 Typical procedure to remove pinacol from obtained products: A vial containing 3,4,5-trimethoxybenzaldehyde (1 mmol, 196 mg) and allenylboronic acid pinacol ester, **1** (1.5 mmol, 250 mg) was irradiated at 300 W at 100 °C for 30 minutes. After this period, the vial contents were dissolved in 50% aqueous MeOH (10 mL) and the contents were transferred to a round-bottomed flask. All volatile materials were removed on a rotary evaporator (45–50 °C/25–15 mbar) and this procedure was repeated until the crude mixture displayed less than 1 mol % of pinacol remaining by gas chromatography analysis [11].

References

1. D. D. Perrin, W. L. F. Armarego, in *Purification of Laboratory Chemicals*, **1980**, Pergamon: Oxford.

2. Li, Q. R.; Gu, C. Z.; Yin, H. *Chin. J. Chem*., **2006**, *24*, 72-78. doi: 10.1002/cjoc.200690024.

3. Chen, J.; Captain, B.; Takenaka, N. *Org. Lett.*, **2011**, *13*, 1654-1657. doi: 10.1021/ol200102c.

4. Freitas, J. J. R.; Couto, T. R.; Cavalcanti, I. H.; Freitas, J. C. R.; Barbosa, Q. P. S.; Oliveira, R. A. *Tetrahedron Lett.*, **2016**, *57*, 760-765, doi: 10.1016/j.tetlet.2016.01.017.

5. Justicia, J.; Sancho-Sanz, I.; Álvarez-Manzaneda, E.; Oltra, J. E.; Cuerva, J. M. *Adv. Synth. Catal.*, **2009**, *351*, 2295-2300. doi: 10.1002/adsc.200900479.

6. Wu, S.; Huang, B.; Gao, X. *Synth. Commun.*, **1990**, *20*, 1279-1286. doi: 10.1080/00397919008052838.

7. Couto, T. R.; Freitas, J. J. R.; Freitas, J. C. R.; Cavalcanti, I. H.; Menezes, P. H; Oliveira, R. A. *Synthesis*, **2015**, *47*, 71-78. doi: 10.1055/s-0034-1379163.

8. Guo, L. N.; Gao, H. J.; Mayer, P.; Knochel, P. *Chem. Eur. J.*, **2010**, *16*, 9829-9834. doi: 10.1002/chem.201000523.

9. Ma, X.; Wang, J. X.; Li, S.; Wang, K. H.; Huang, D. *Tetrahedron*, **2009**, *65*, 8683-8689. doi: 10.1016/j.tet.2009.08.051.

10. Lennox, A. J. J.; Lloyd-Jones, G. C.; *Angew. Chem. Int. Ed.*, **2012**, *51*, 9385-9388. doi: 10.1002/anie.201203930.

11. Nowrouzi, F.; Batey, R. A.; *Angew. Chem. Int. Ed.,* **2013**, *52*, 892-895. doi: 10.1002/anie.201207978.

12. Bagutski, V.; Ros, A.; Aggarwal, V. K.; *Tetrahedron*, **2009**, *65*, 9956-9960. doi: 10.1016/j.tet.2009.10.002.

3. Spectra

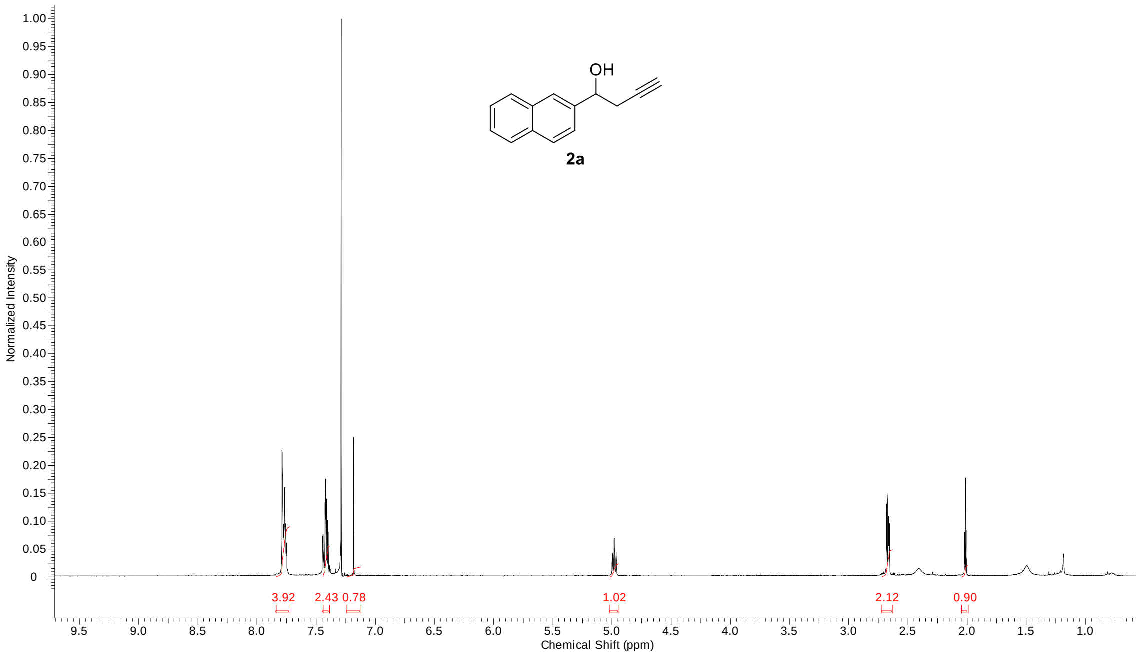

^{1}H NMR spectrum (300 MHz, $CDCl_3$) of compound **2a**

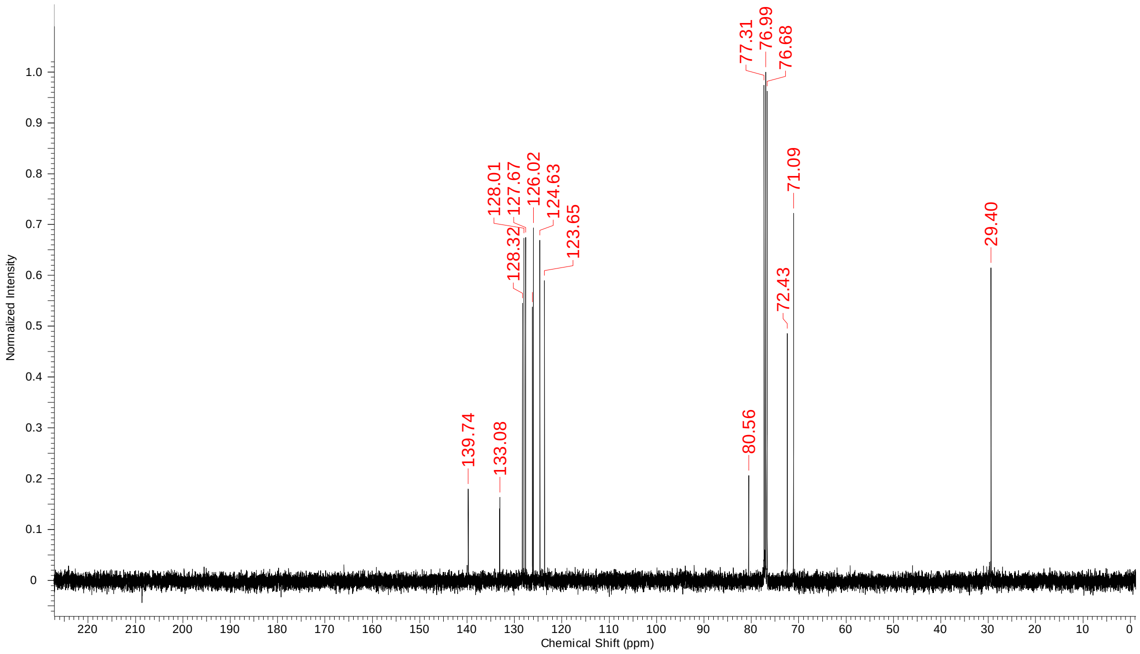

^{13}C NMR spectrum (75 MHz, $CDCl_3$) of compound **2a**

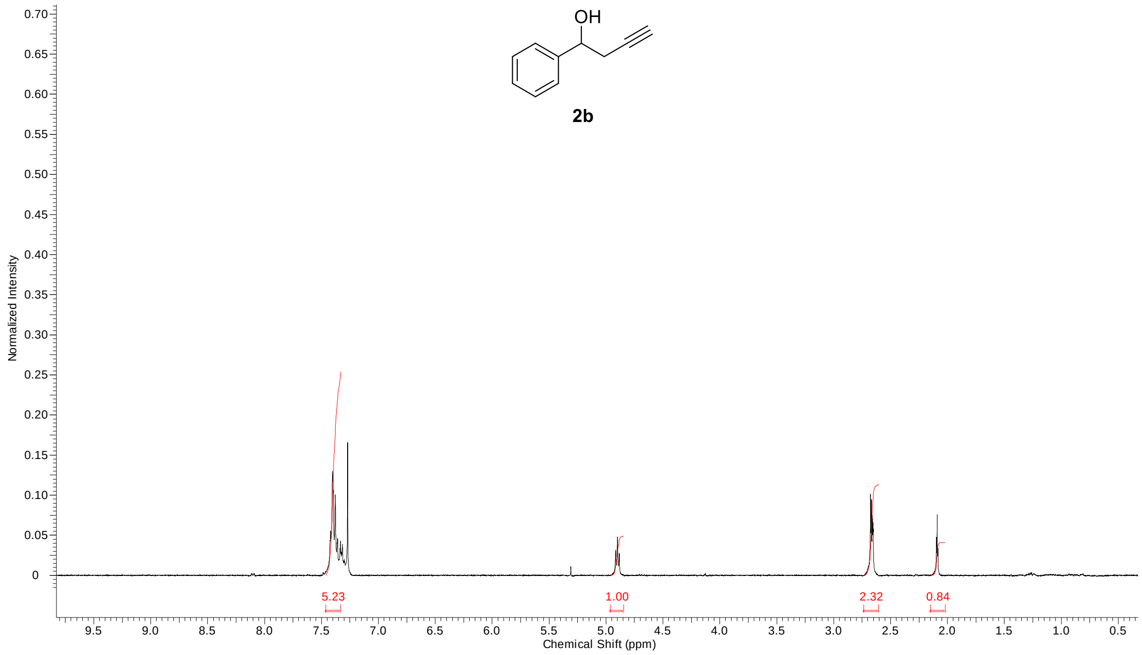

^{1}H NMR spectrum (300 MHz, $CDCl_3$) of compound **2b**

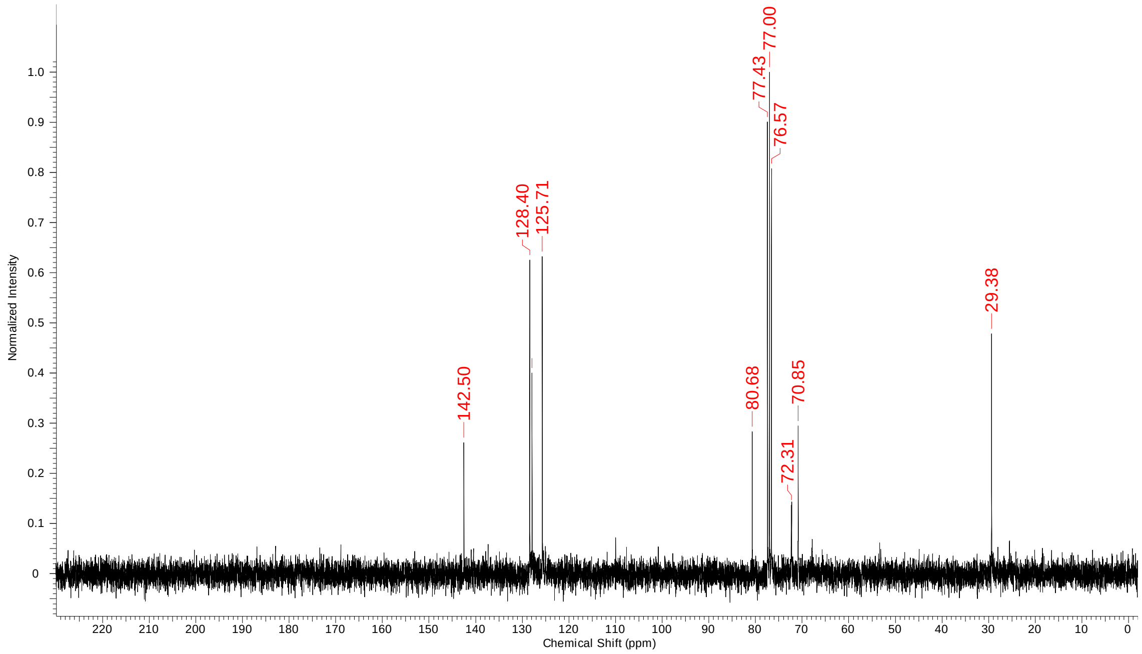

^{13}C NMR spectrum (75 MHz, $CDCl_3$) of compound **2b**

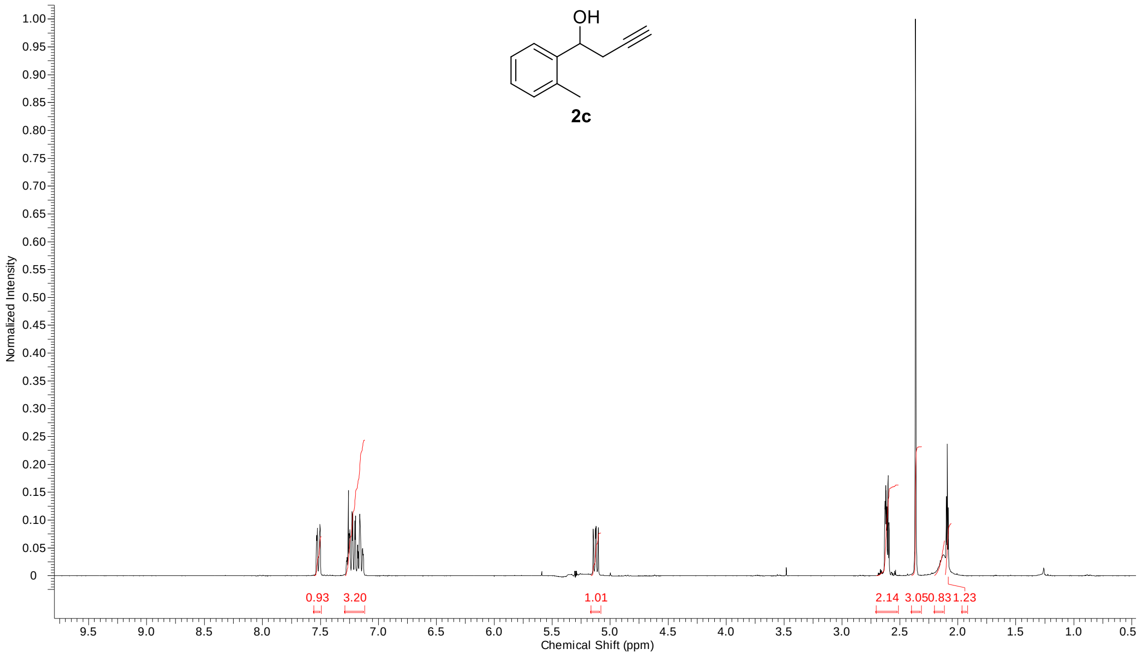

^{1}H NMR spectrum (300 MHz, $CDCl_3$) of compound **2c**

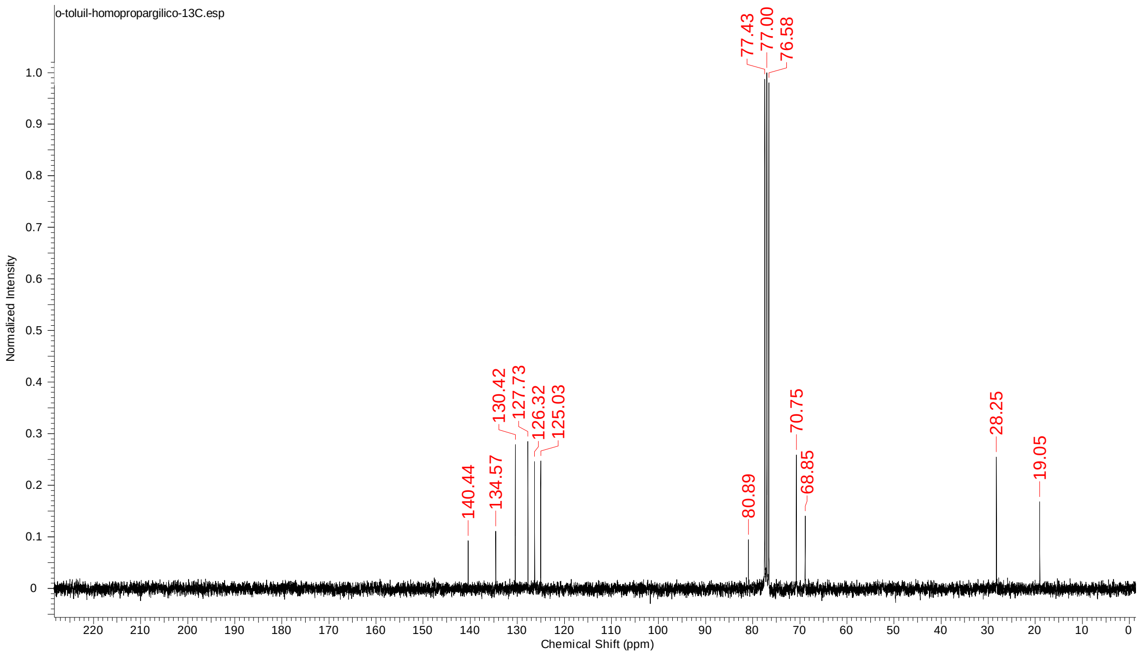

^{13}C NMR spectrum (75 MHz, $CDCl_3$) of compound **2c**

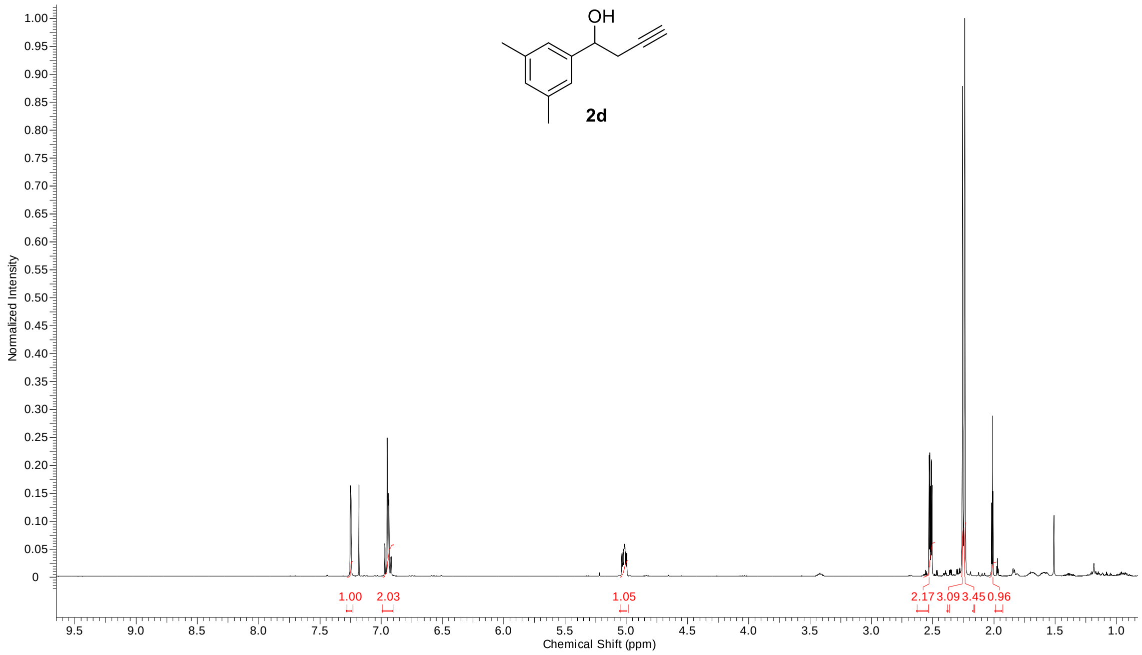

^{1}H NMR spectrum (300 MHz, $CDCl_3$) of compound **2d**

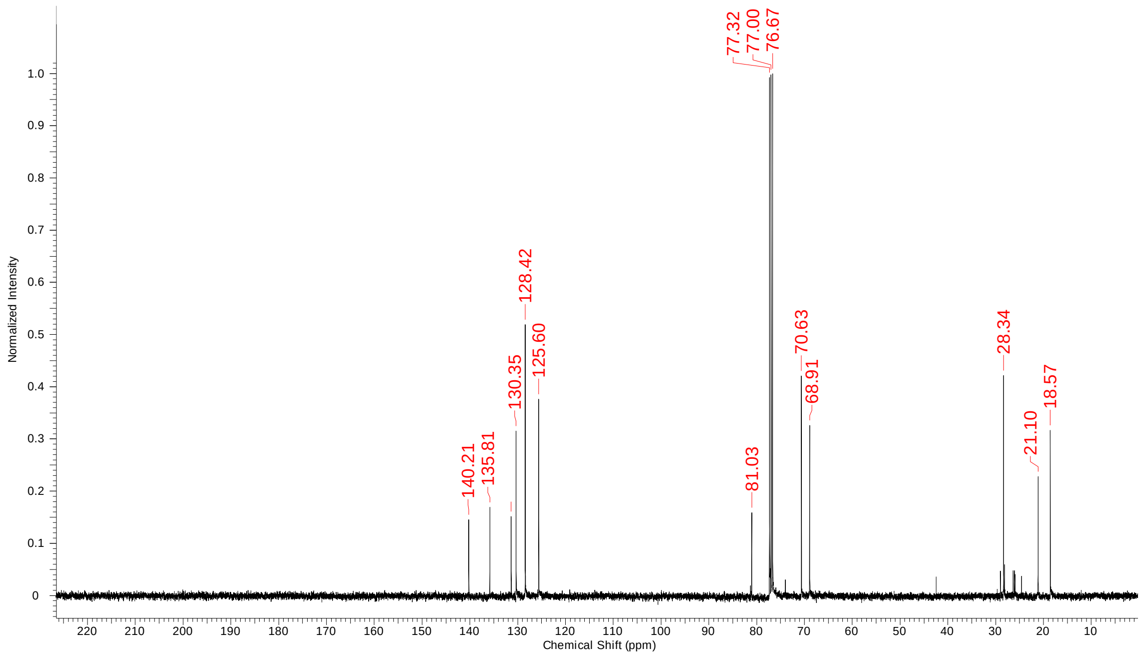

^{13}C NMR spectrum (75 MHz, $CDCl_3$) of compound **2d**

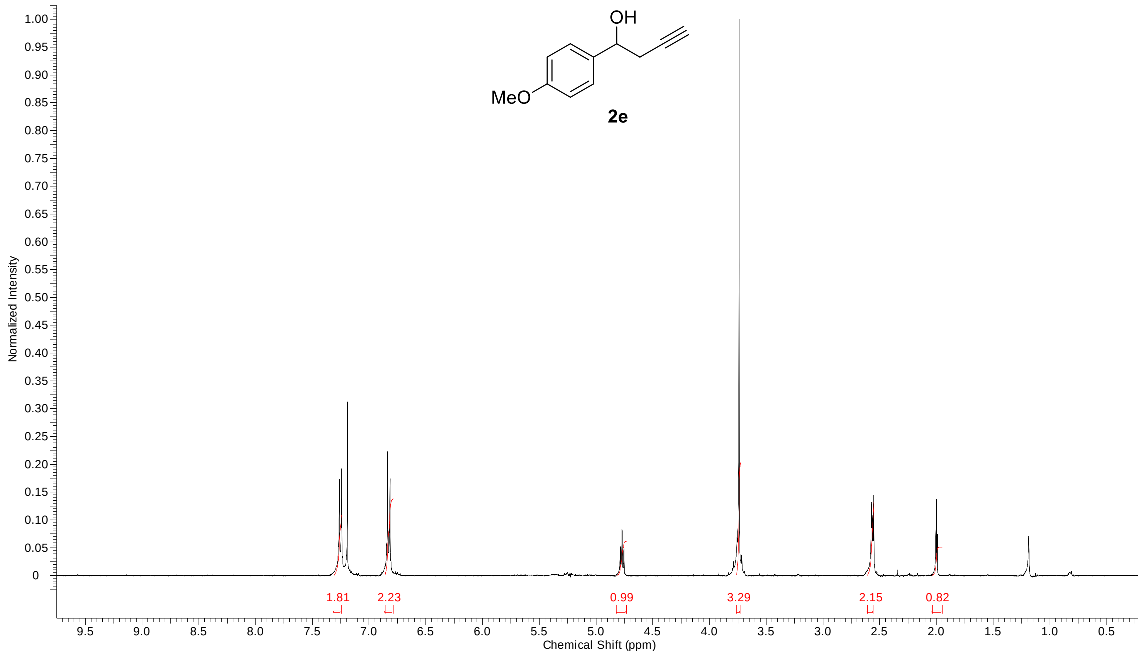

^{1}H NMR spectrum (300 MHz, $CDCl_3$) of compound **2e**

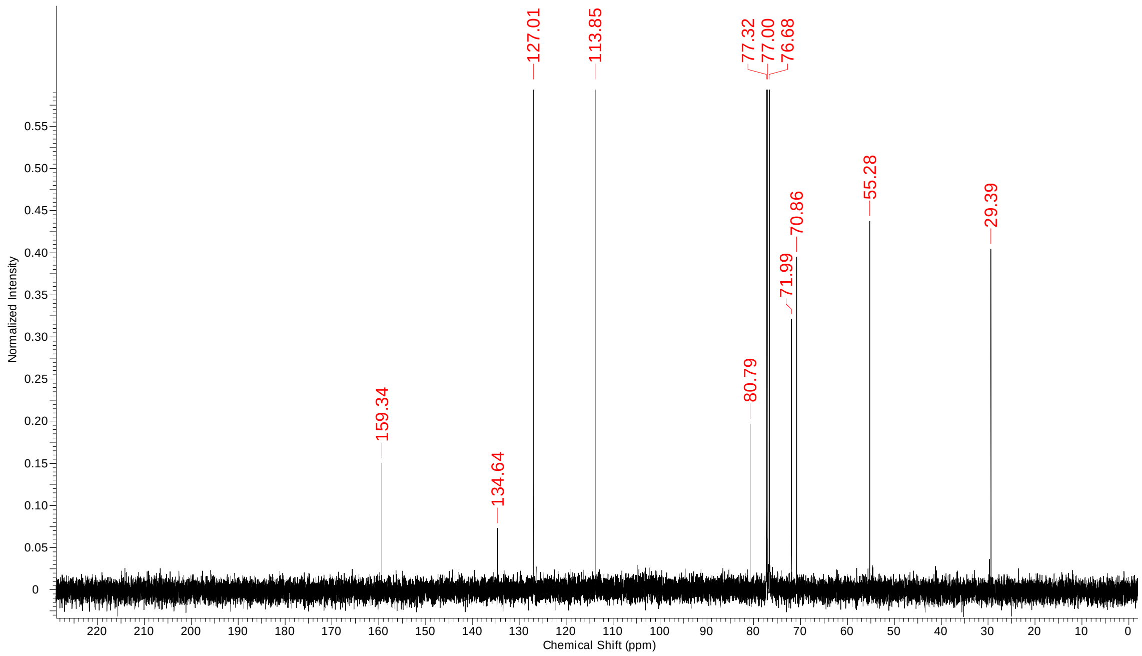

^{13}C NMR spectrum (75 MHz, $CDCl_3$) of compound **2e**

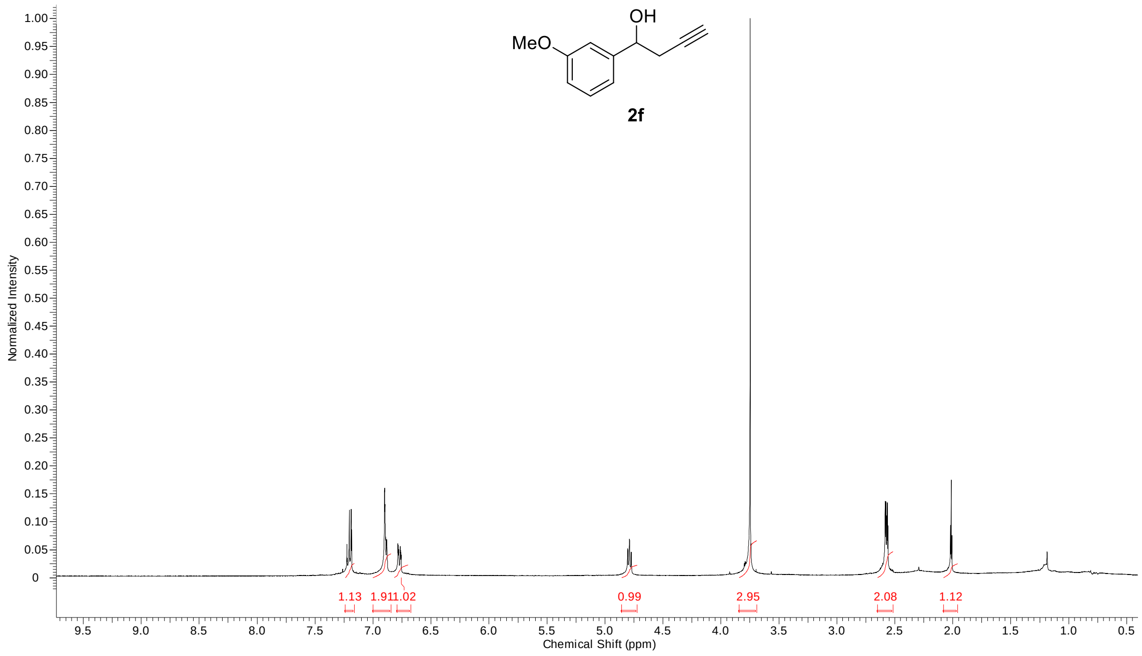

^{1}H NMR spectrum (300 MHz, $CDCl_3$) of compound **2f**

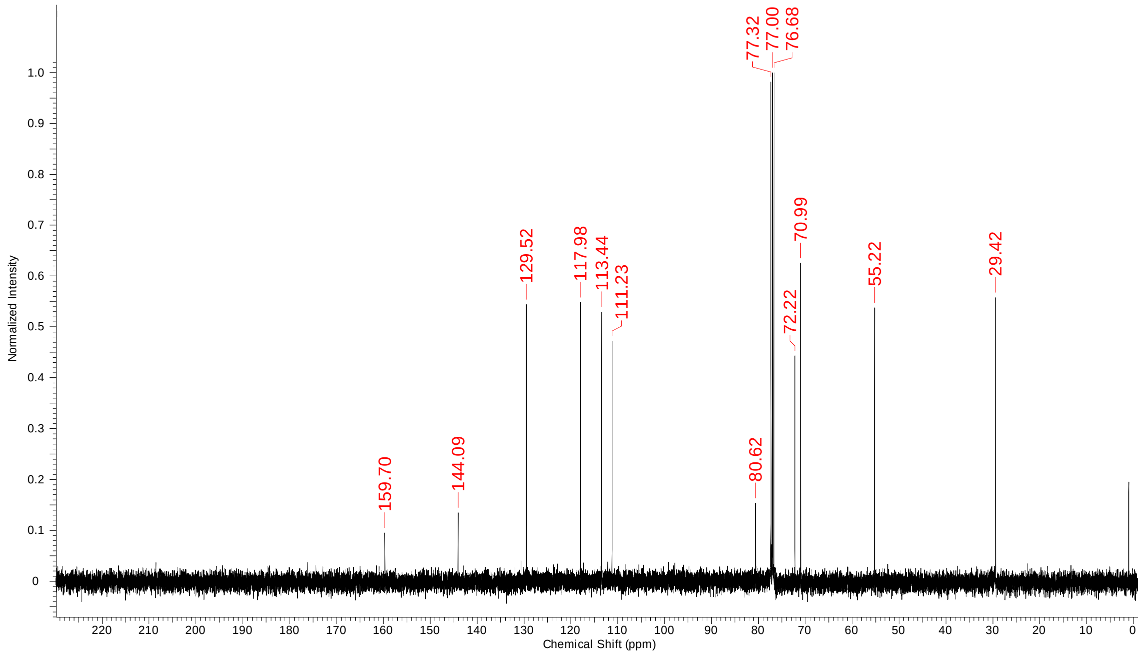

^{13}C NMR spectrum (75 MHz, $CDCl_3$) of compound **2f**

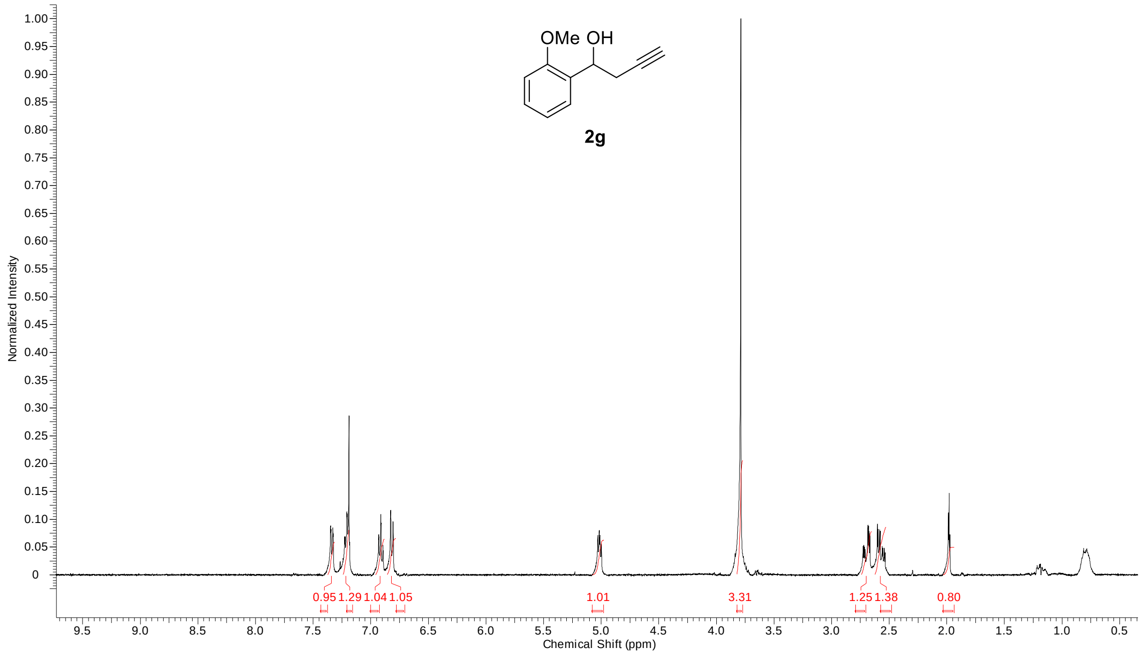

^{1}H NMR spectrum (300 MHz, CDCl$_3$) of compound **2g**

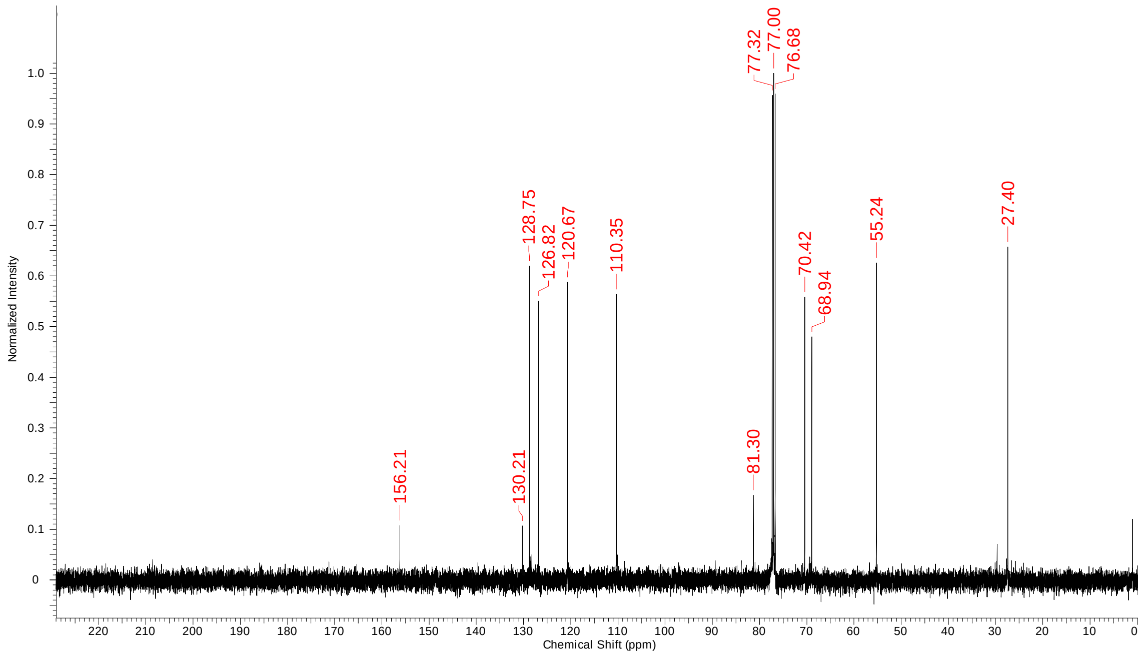

^{13}C NMR spectrum (75 MHz, CDCl$_3$) of compound **2g**

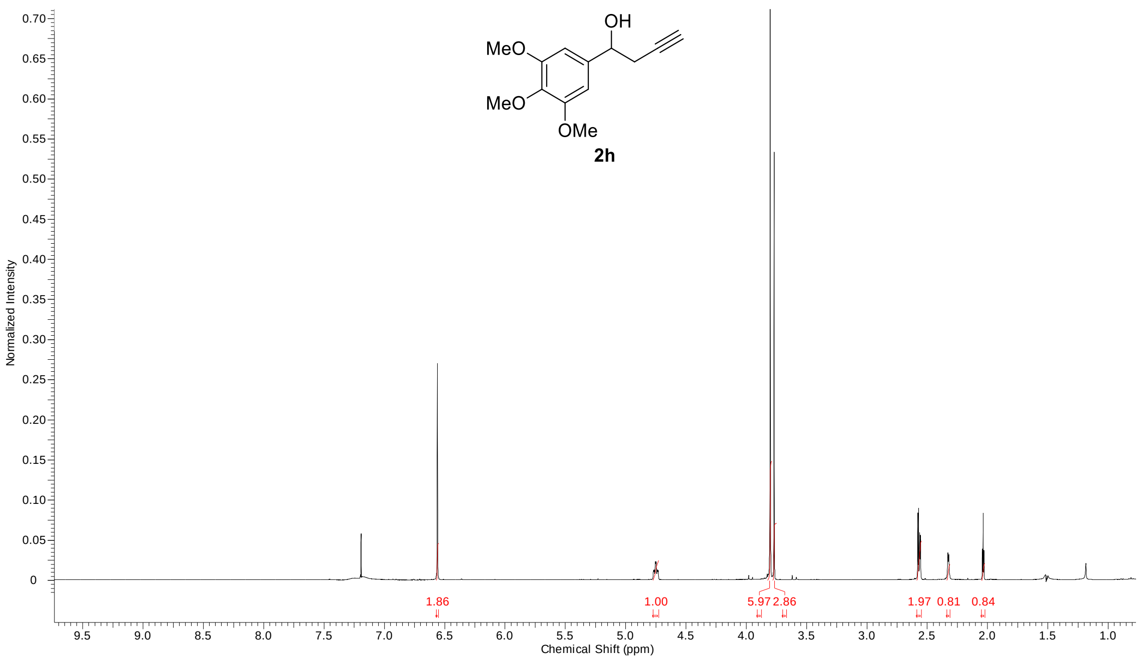

^{1}H NMR spectrum (300 MHz, $CDCl_3$) of compound **2h**

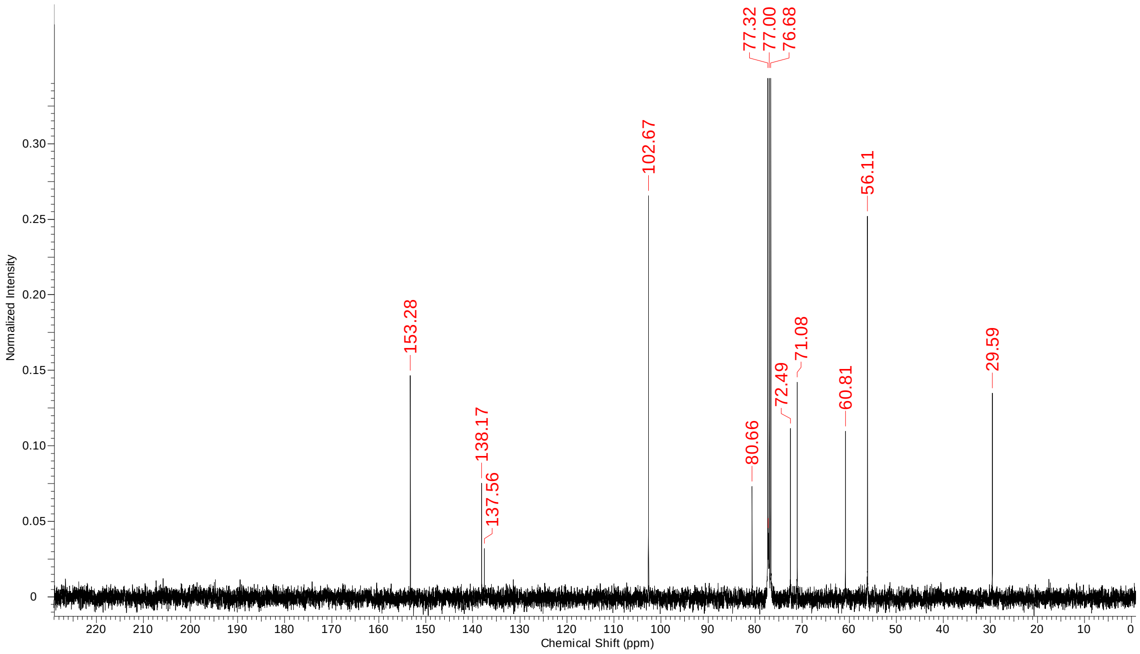

^{13}C NMR spectrum (75 MHz, $CDCl_3$) of compound **2h**

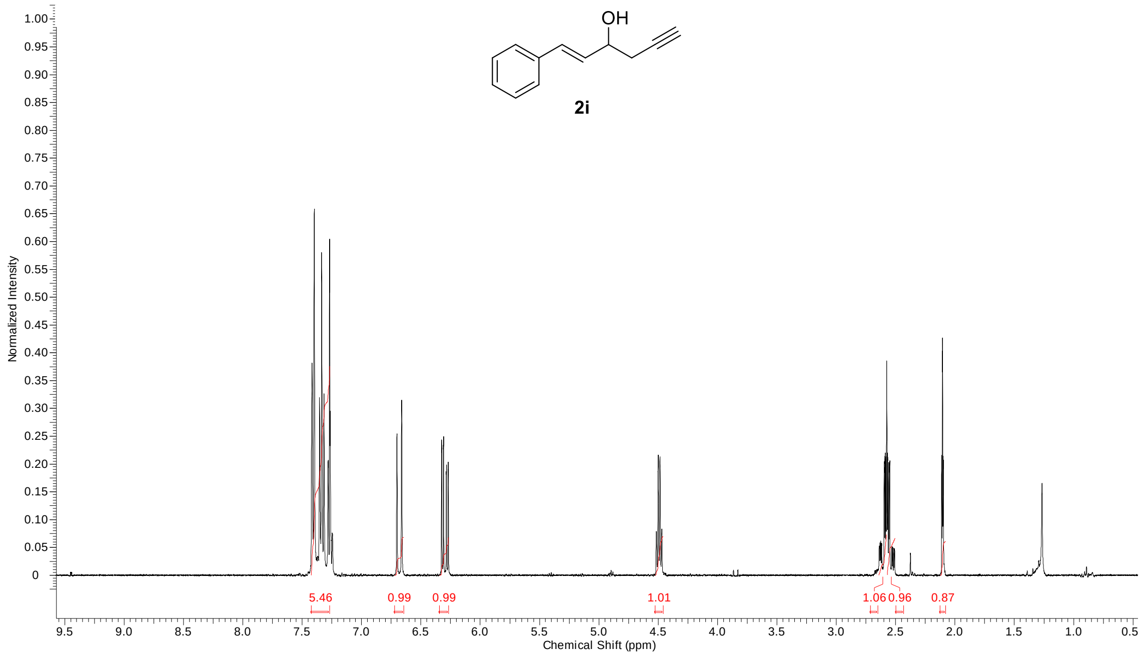

^{1}H NMR spectrum (300 MHz, $CDCl_3$) of compound **2i**

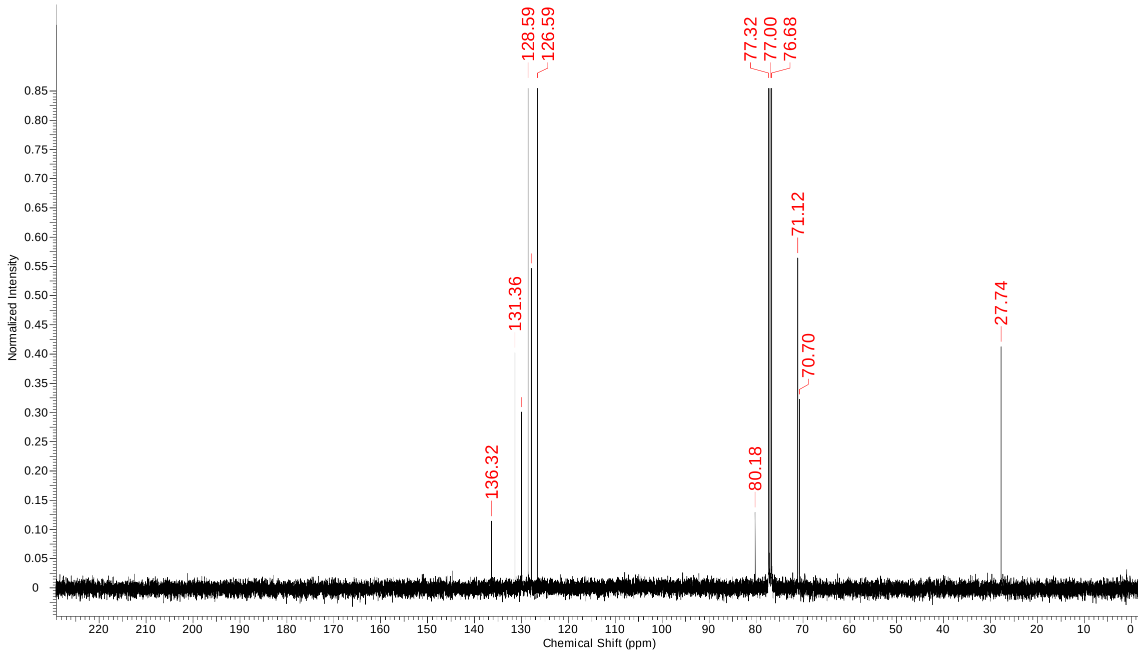

^{13}C NMR spectrum (75 MHz, $CDCl_3$) of compound **2i**

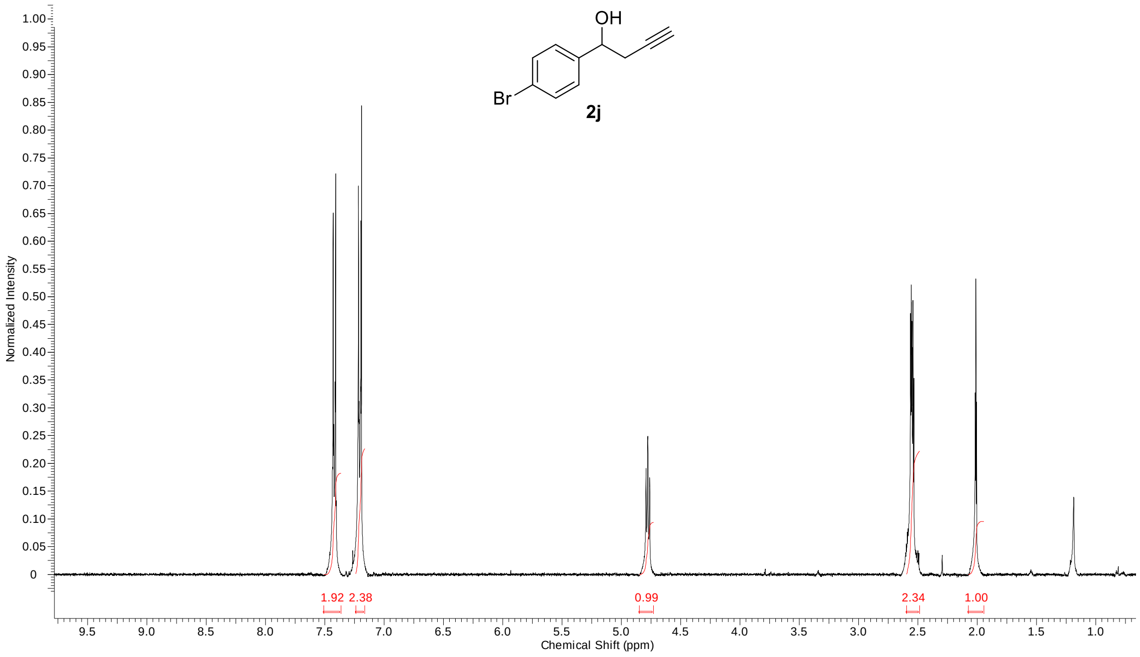

^{1}H NMR spectrum (300 MHz, $CDCl_3$) of compound **2j**

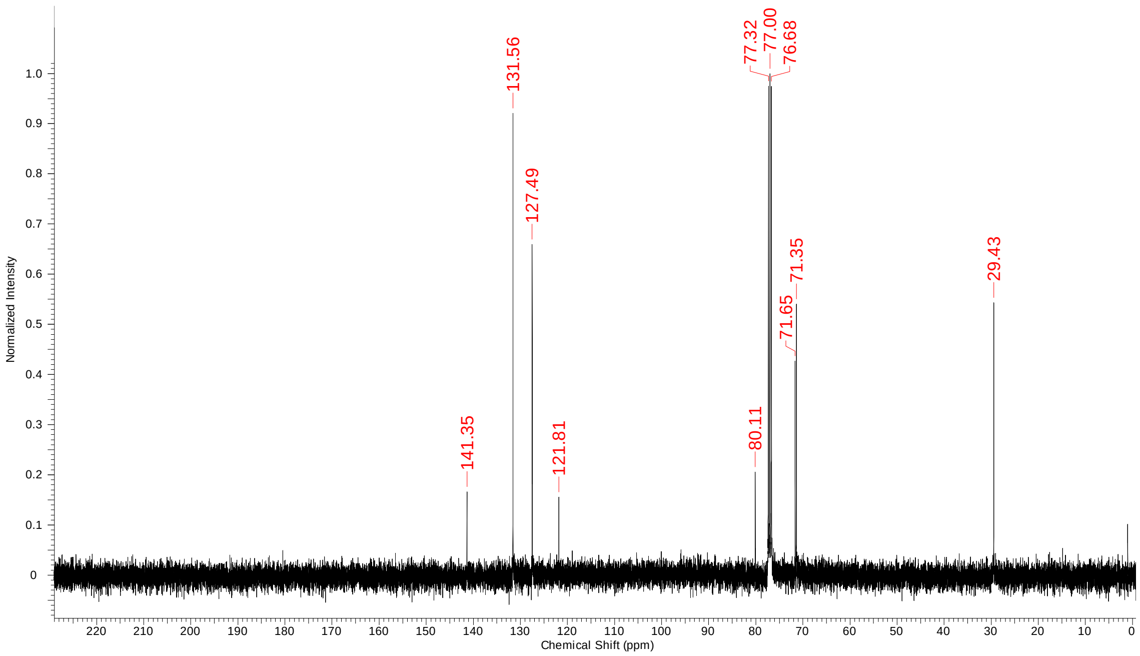

^{13}C NMR spectrum (75 MHz, $CDCl_3$) of compound **2j**

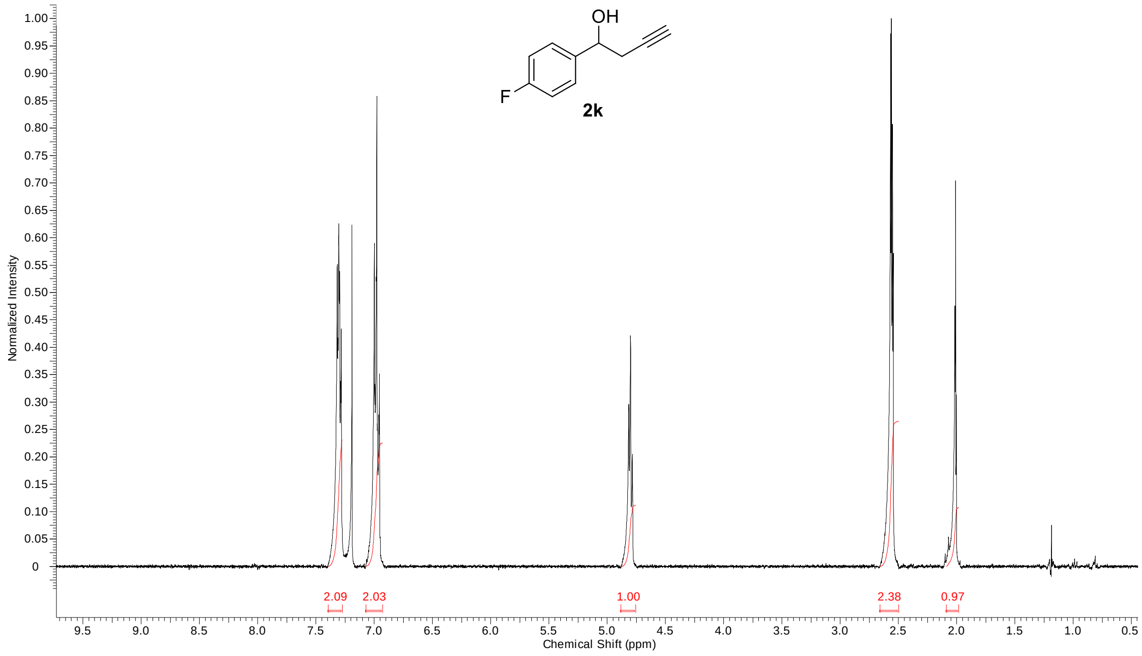

^{1}H NMR spectrum (300 MHz, $CDCl_3$) of compound **2k**

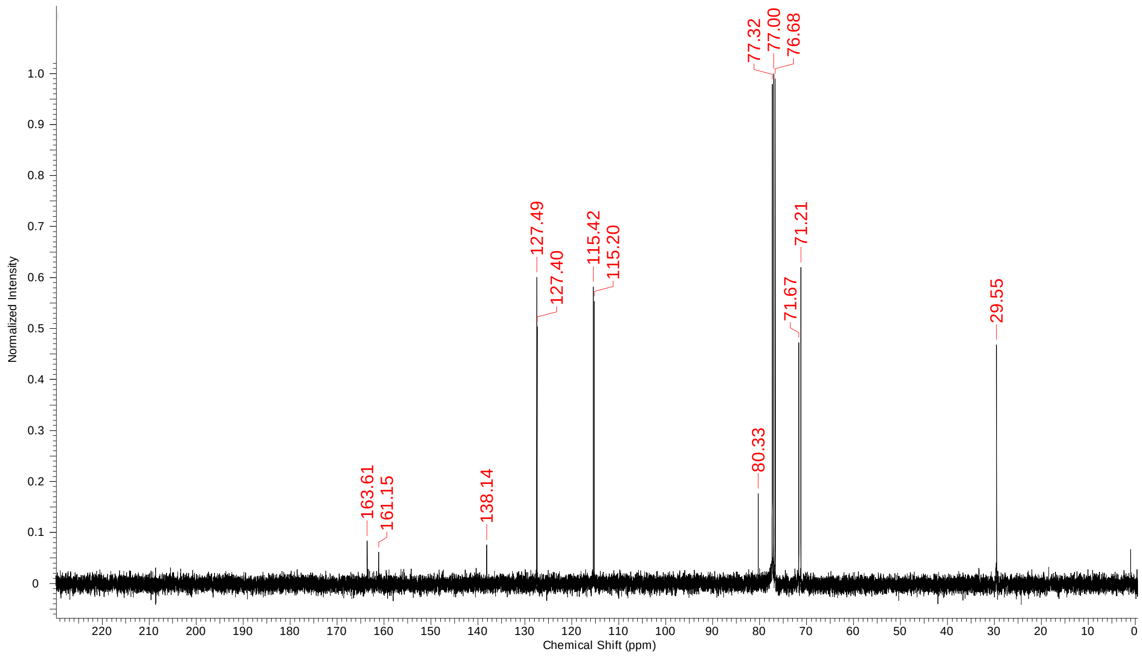

^{13}C NMR spectrum (75 MHz, $CDCl_3$) of compound **2k**

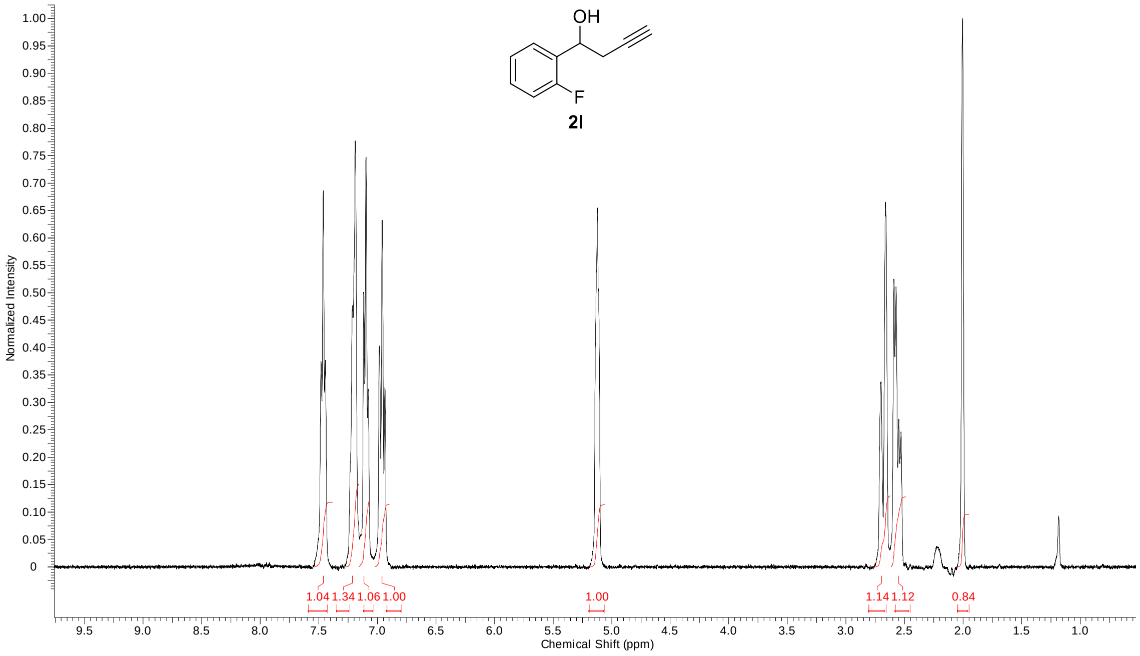

^{1}H NMR spectrum (300 MHz, $CDCl_3$) of compound **2l**

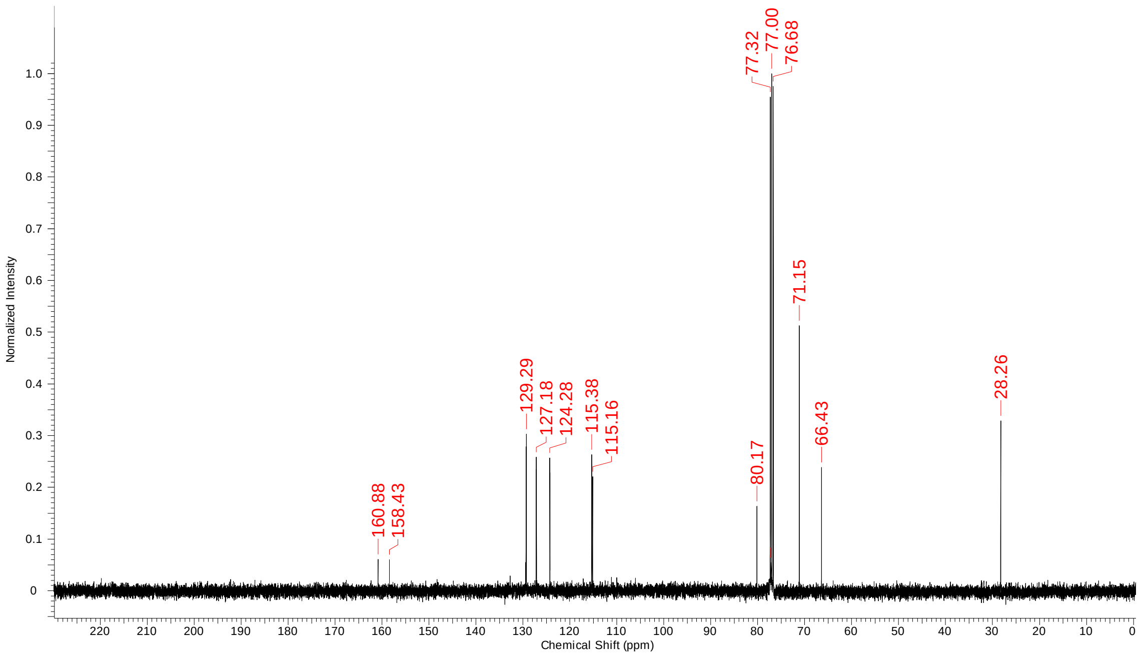

^{13}C NMR spectrum (75 MHz, $CDCl_3$) of compound **2l**

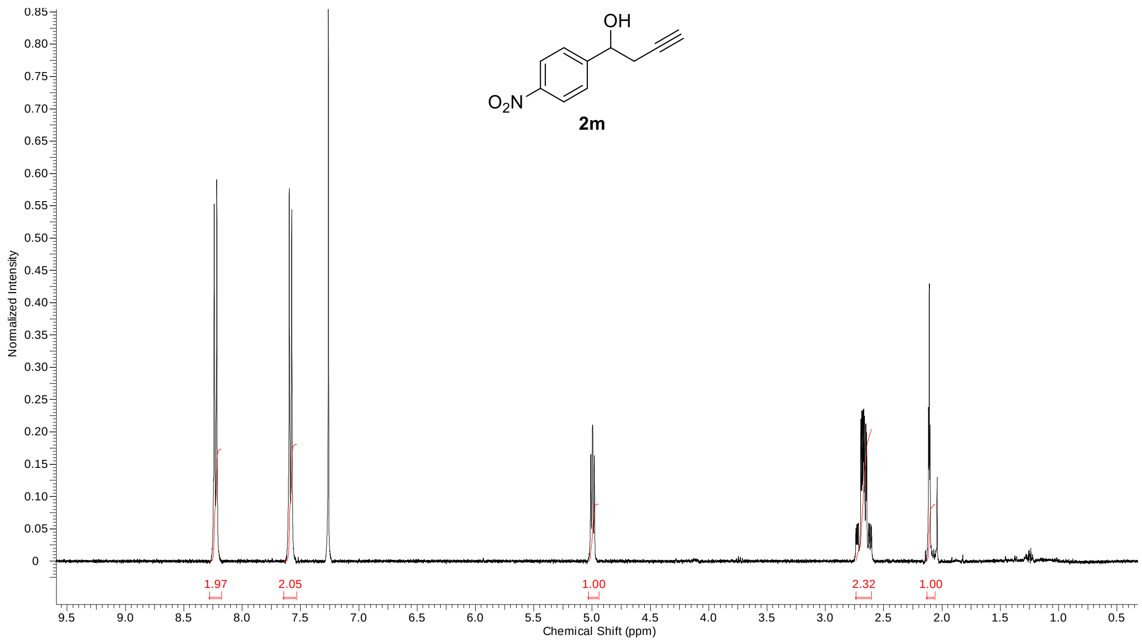

^1H NMR spectrum (300 MHz, $CDCl_3$) of compound **2m**

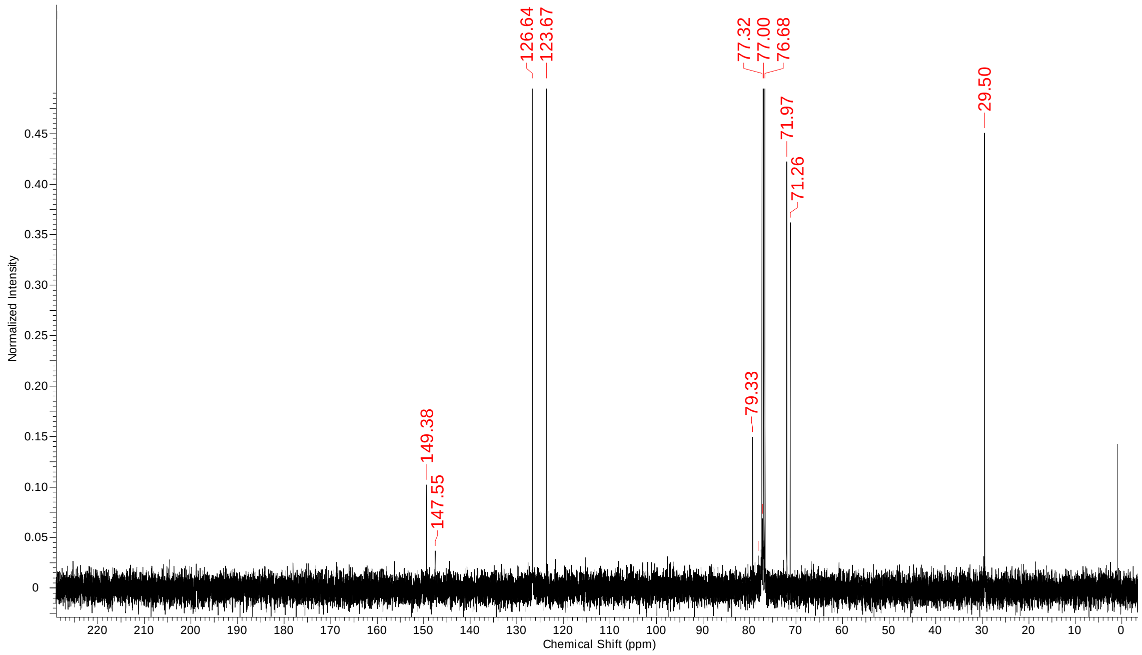

^{13}C NMR spectrum (75 MHz, $CDCl_3$) of compound **2m**

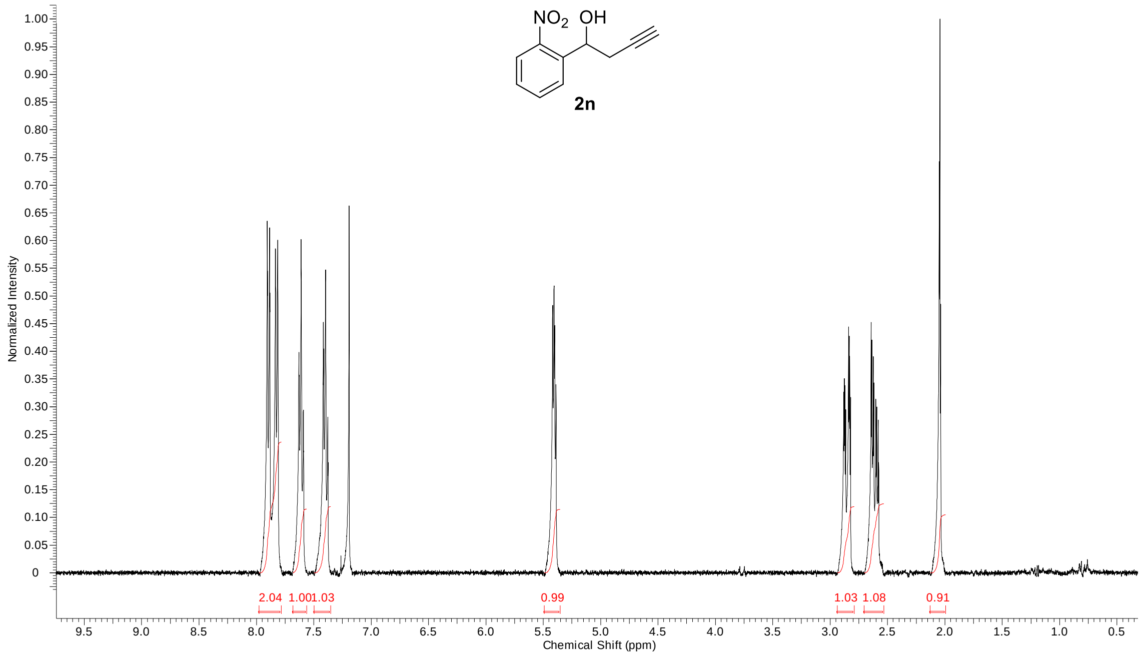

^{1}H NMR spectrum (300 MHz, $CDCl_3$) of compound **2n**

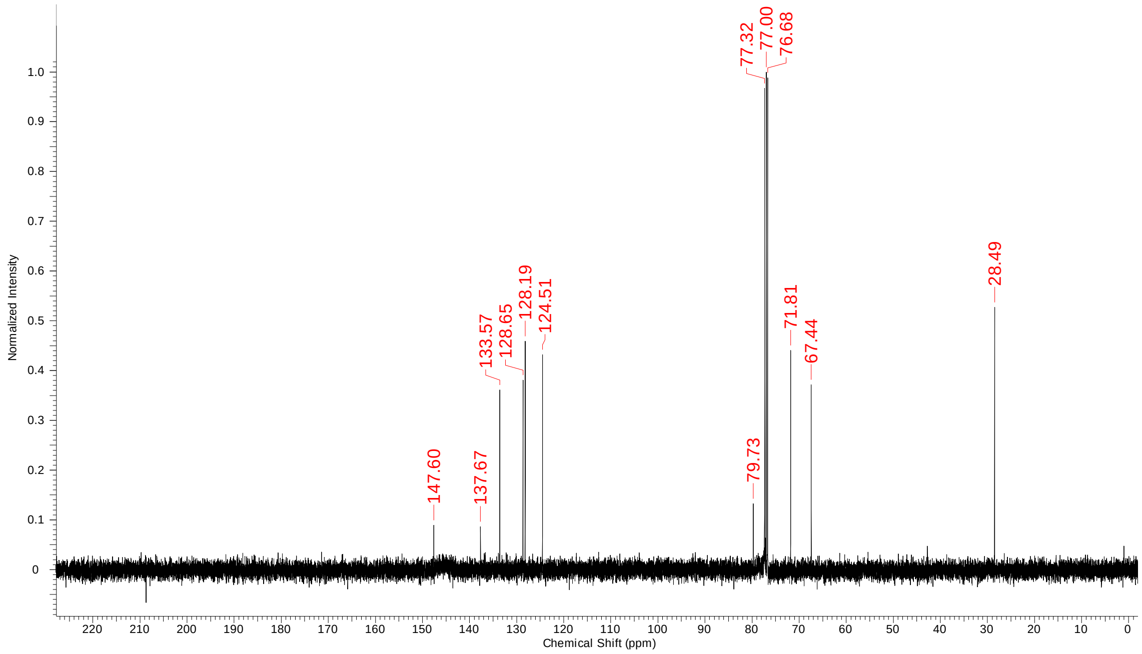

^{13}C NMR spectrum (75 MHz, $CDCl_3$) of compound **2n**

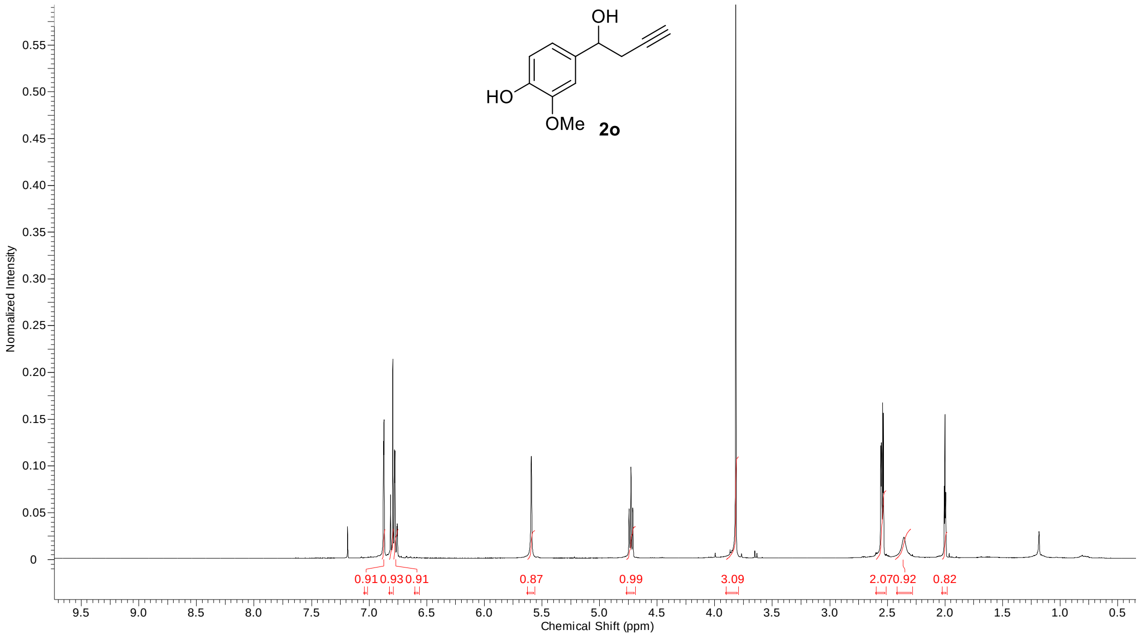

^{1}H NMR spectrum (300 MHz, $CDCl_3$) of compound **2o**

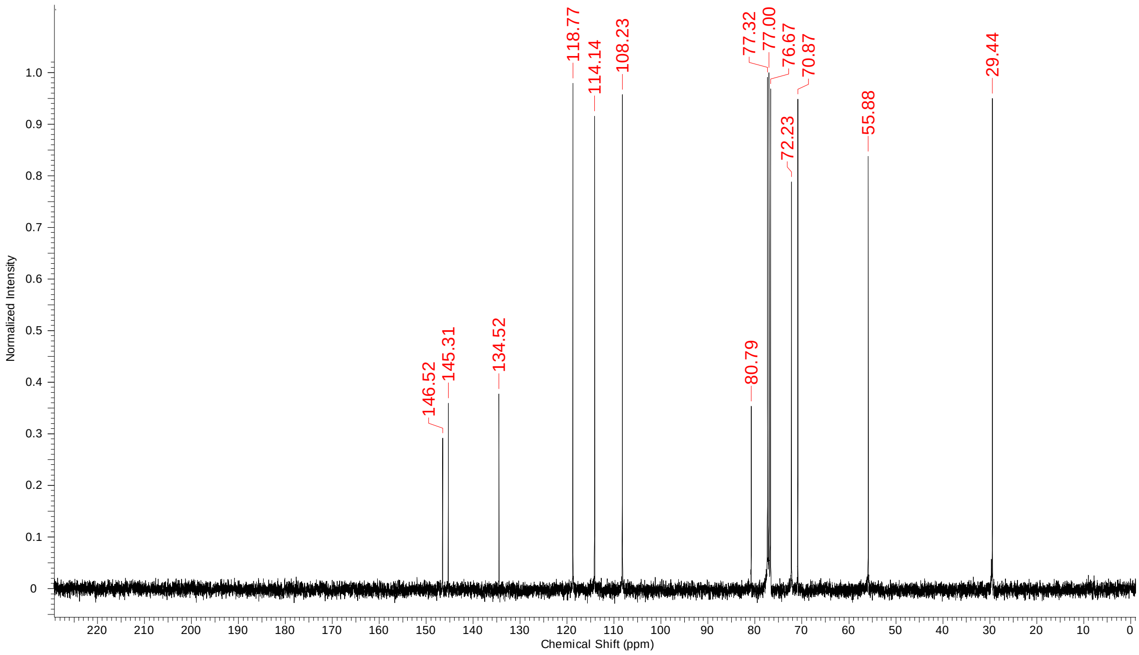

^{13}C NMR spectrum (75 MHz, $CDCl_3$) of compound **2o**

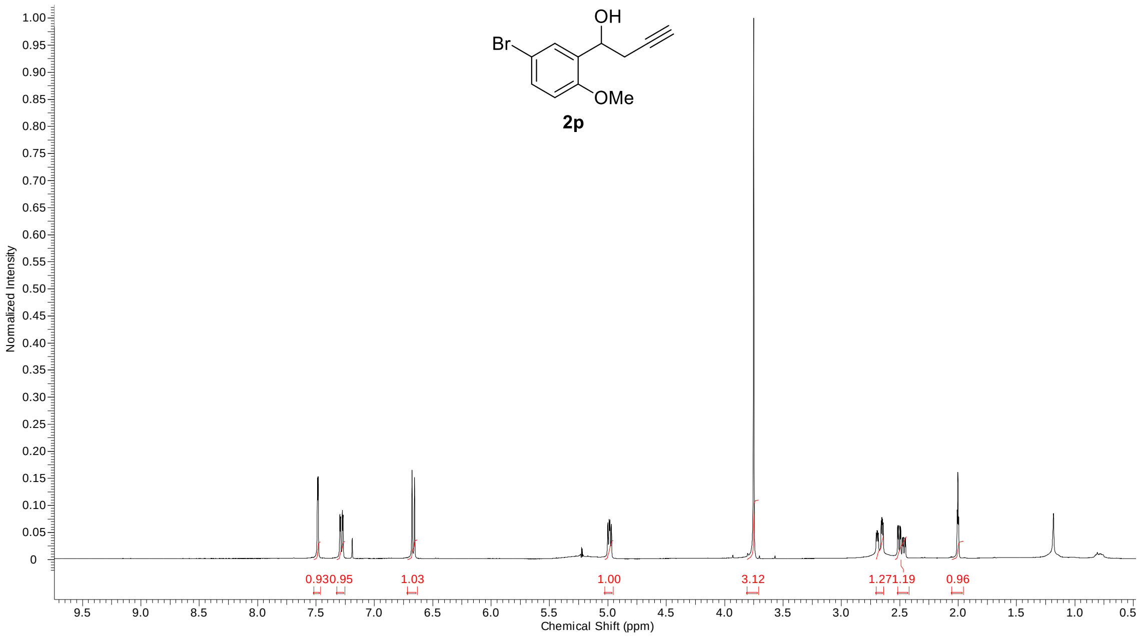

^{1}H NMR spectrum (300 MHz, $CDCl_3$) of compound **2p**

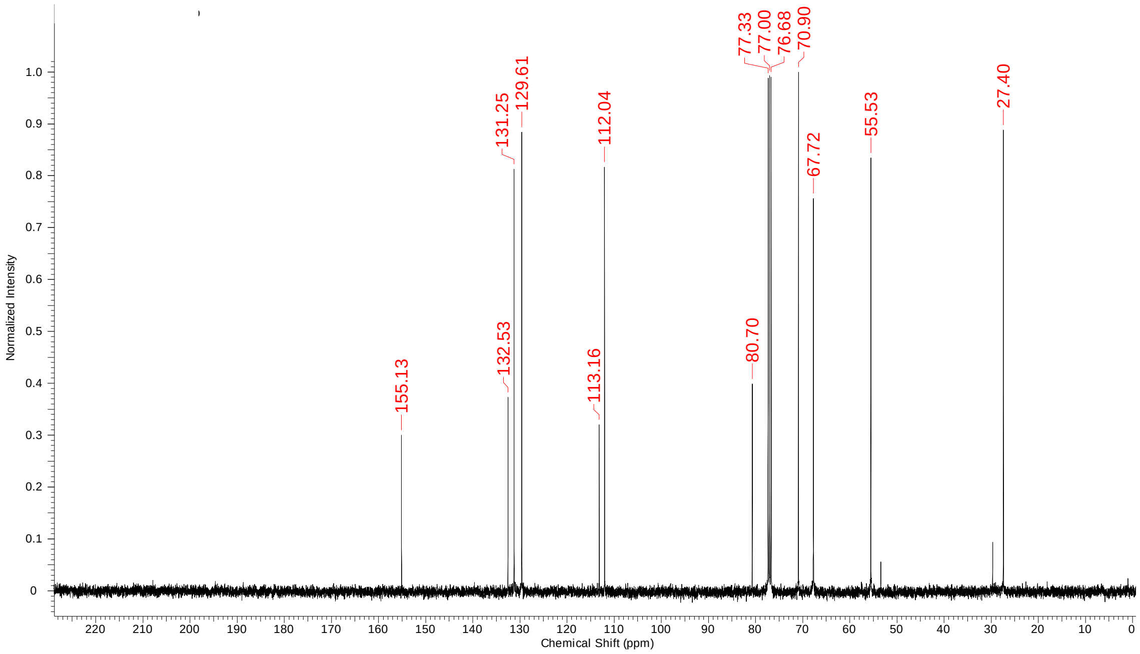

^{13}C NMR spectrum (75 MHz, $CDCl_3$) of compound **2p**

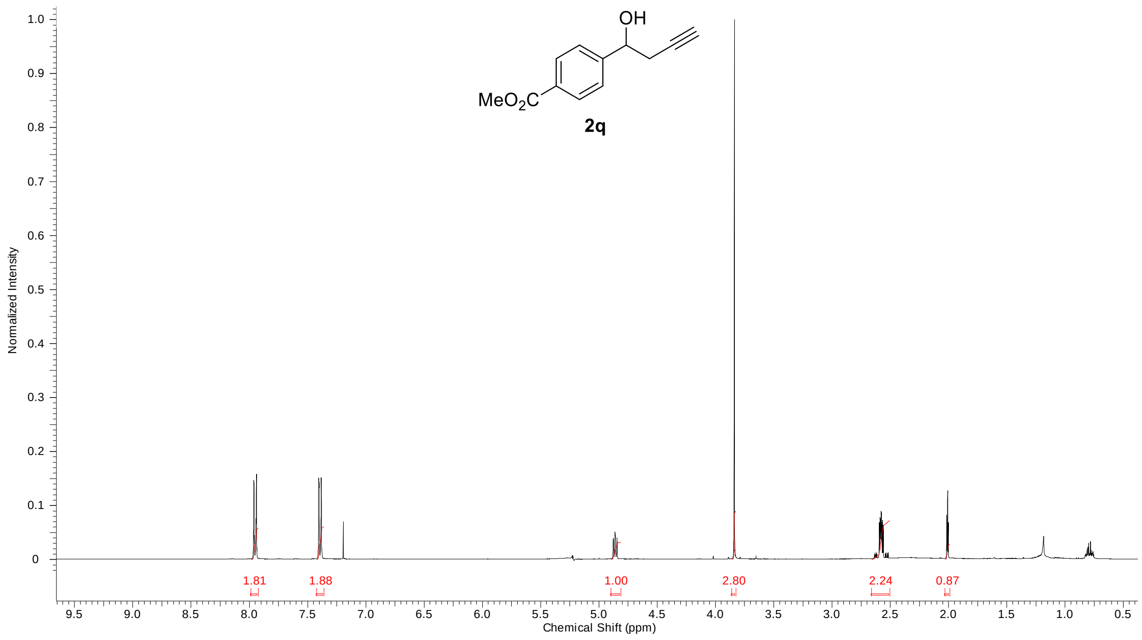

^{1}H NMR spectrum (300 MHz, $CDCl_3$) of compound **2q**

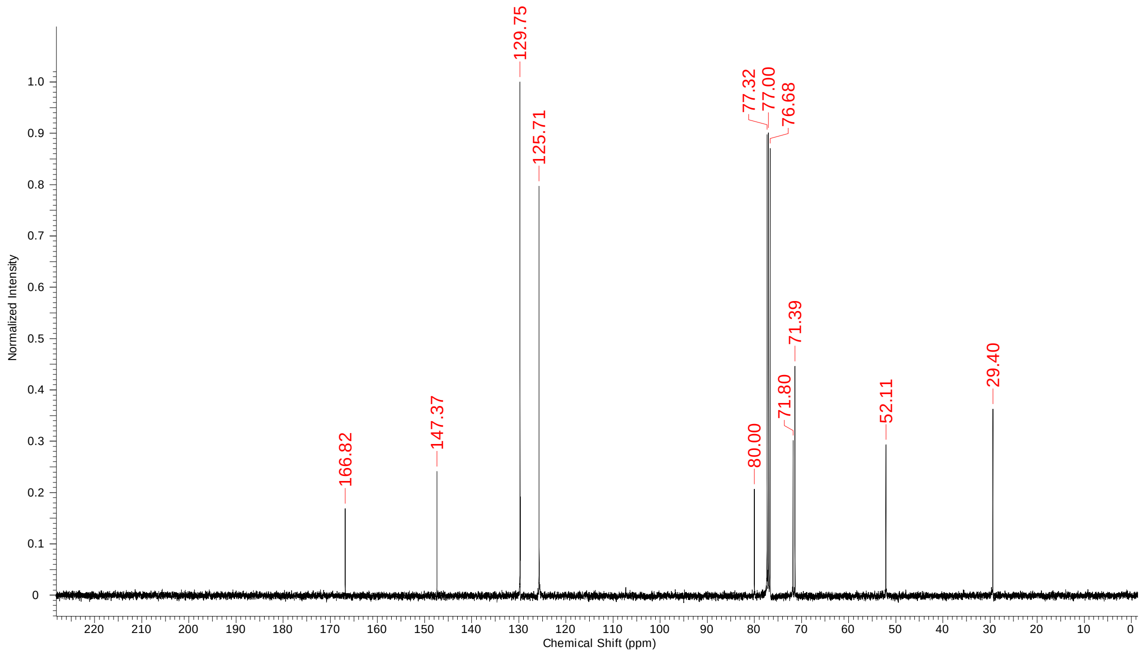

^{13}C NMR spectrum (75 MHz, $CDCl_3$) of compound **2q**

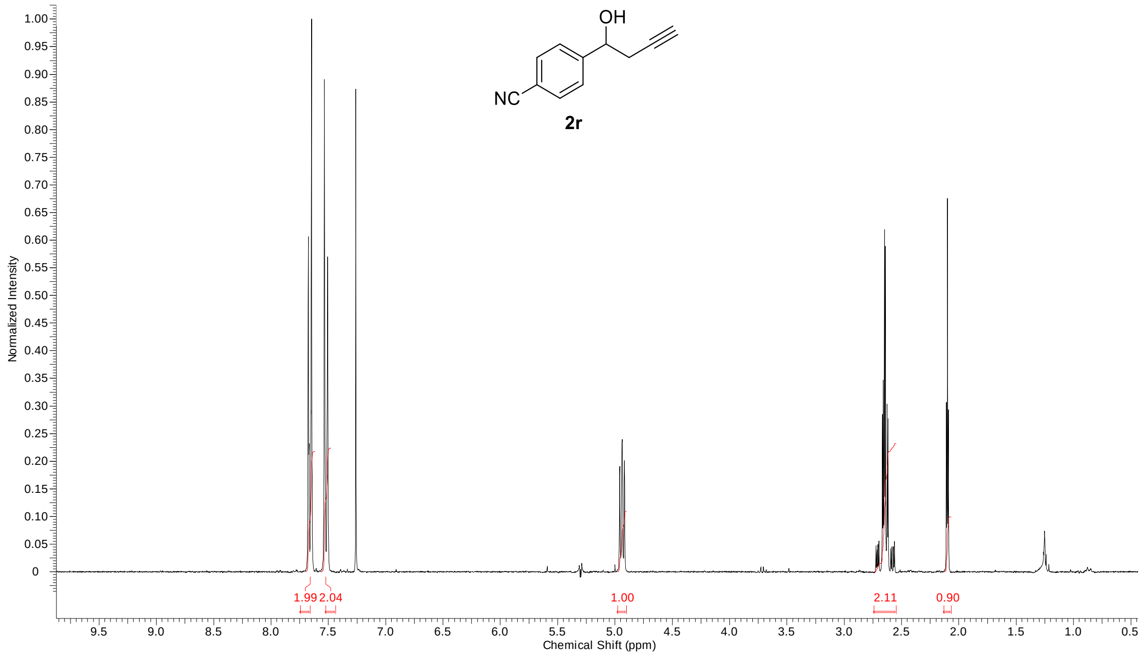

^{1}H NMR spectrum (300 MHz, $CDCl_3$) of compound **2r**

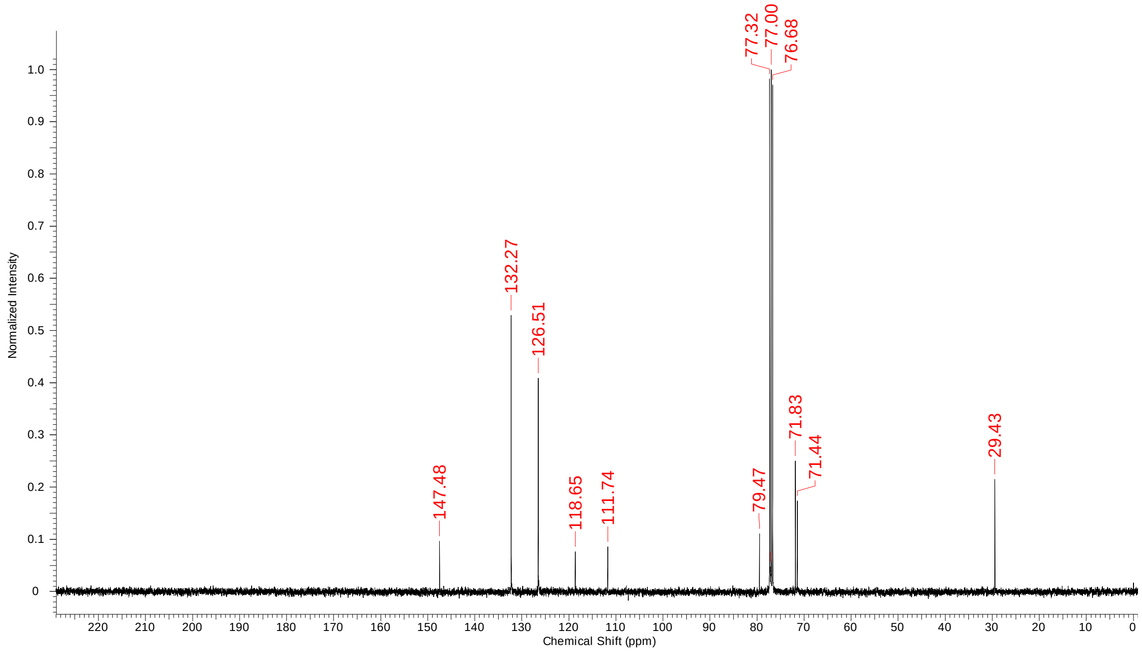

^{13}C NMR spectrum (75 MHz, $CDCl_3$) of compound **2r**

L1101_11

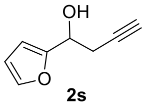

2s

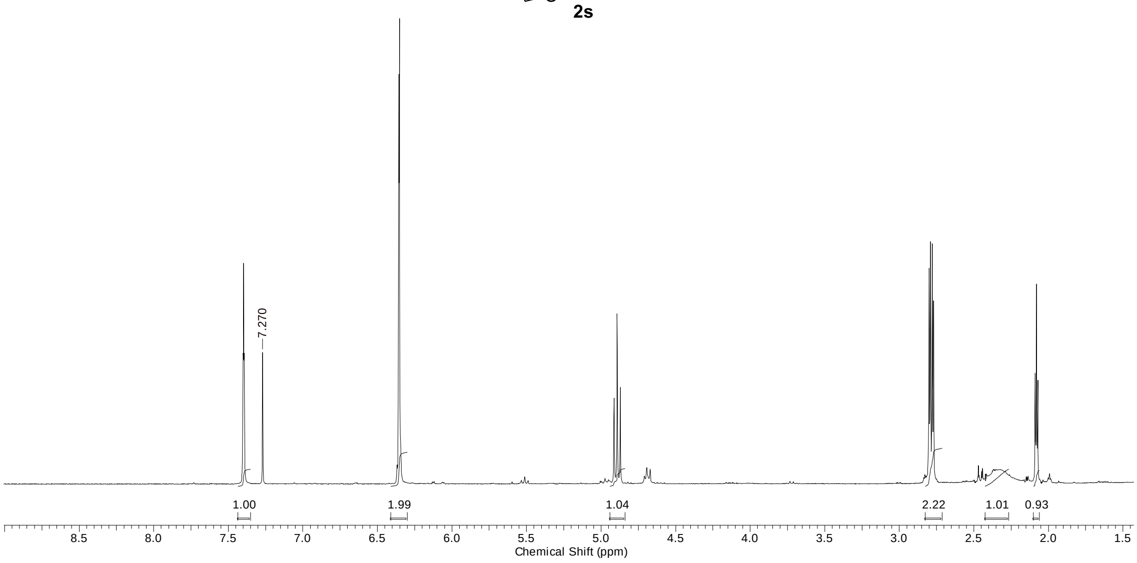

^{1}H NMR spectrum (300 MHz, $CDCl_3$) of compound **2s**

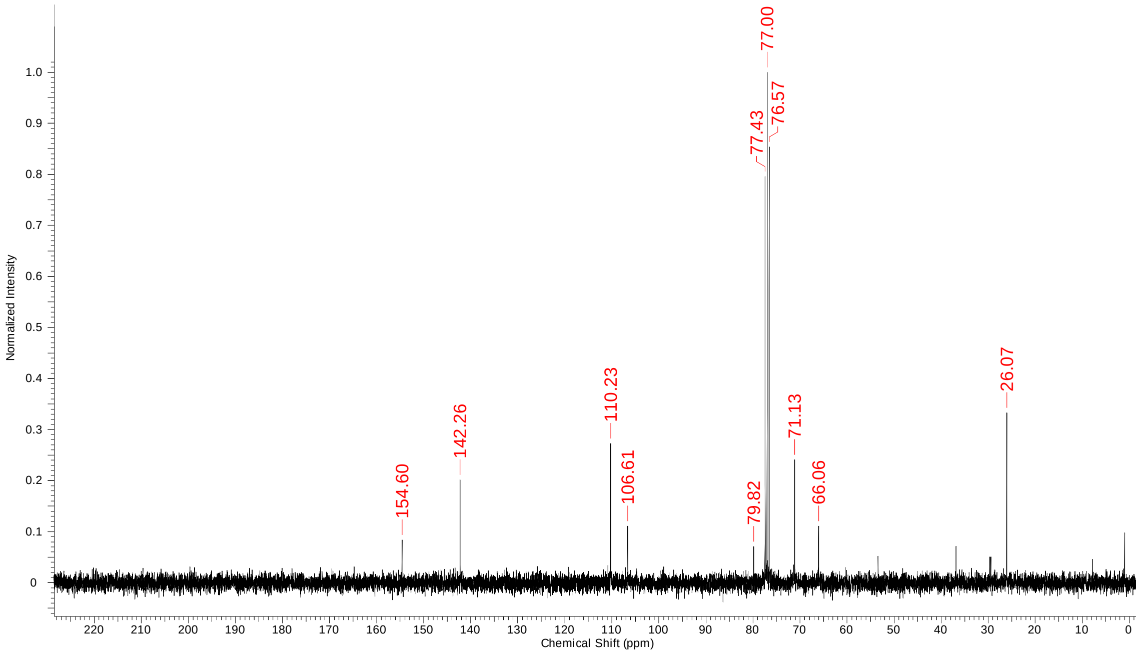

^{13}C NMR spectrum (75 MHz, $CDCl_3$) of compound **2s**

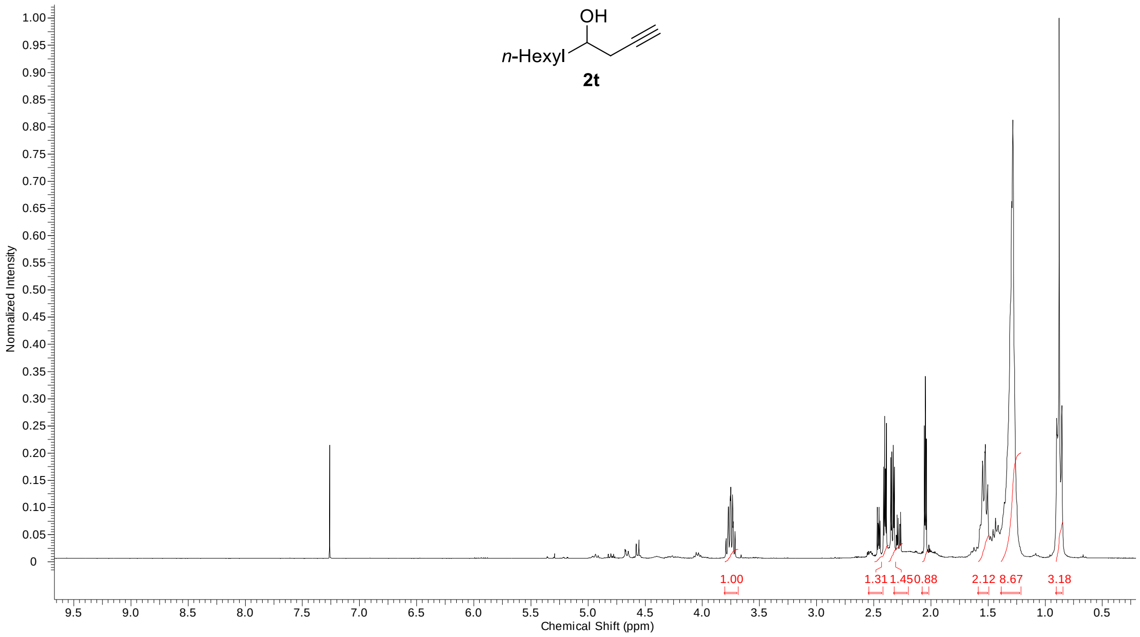

^{1}H NMR spectrum (300 MHz, $CDCl_3$) of compound **2t**

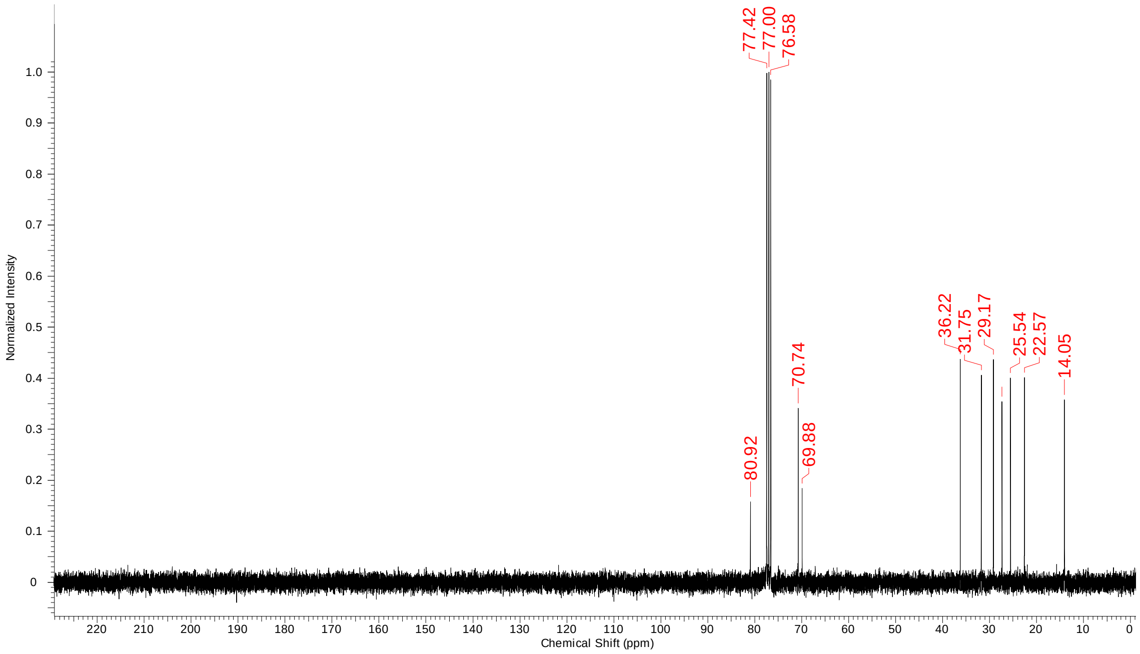

^{13}C NMR spectrum (75 MHz, $CDCl_3$) of compound **2t**

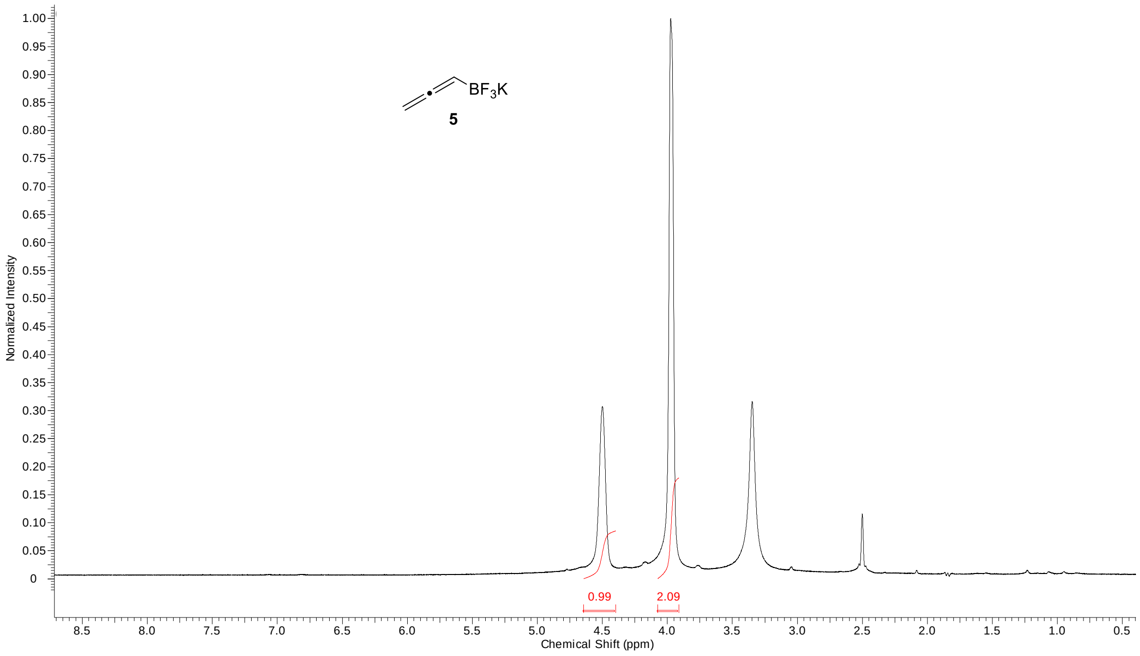

^{1}H NMR spectrum (400 MHz, DMSO-d_6) of compound **4**

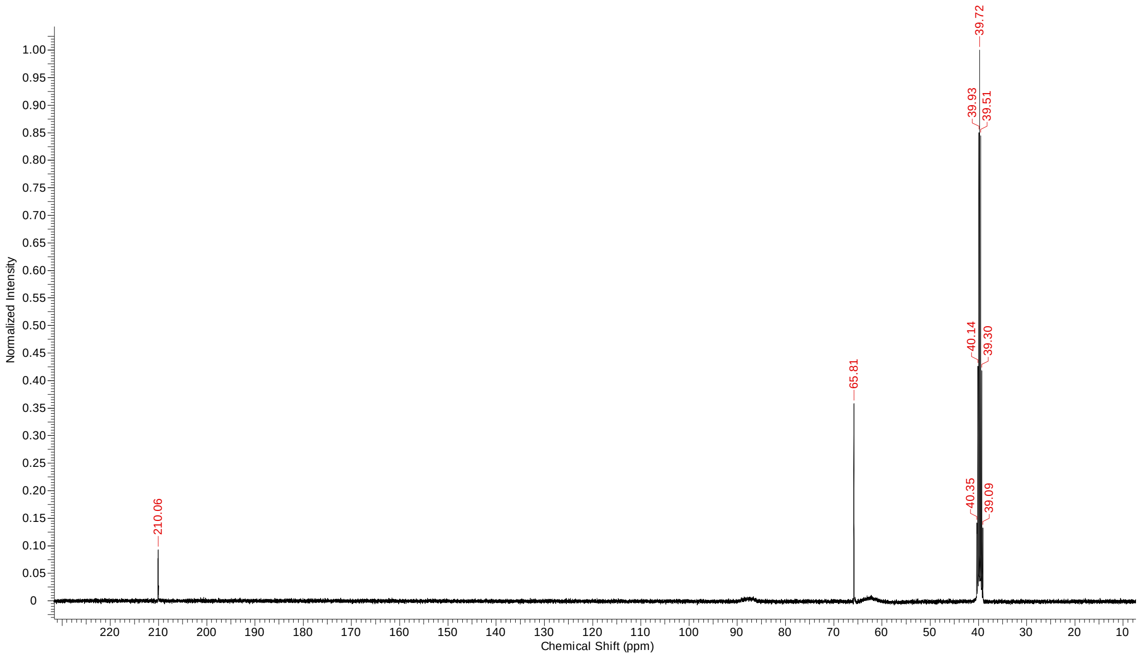

^{13}C NMR spectrum (100 MHz, DMSO-d_6) of compound **4**

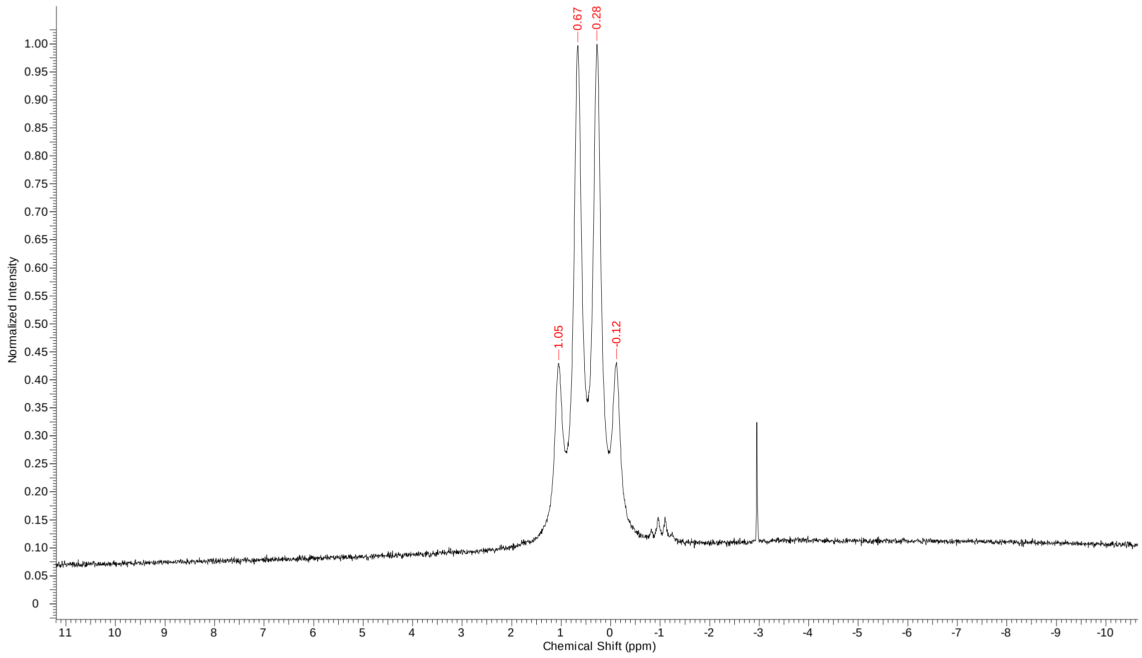

^{19}F NMR spectrum (376 MHz, DMSO-d_6) of compound **4**

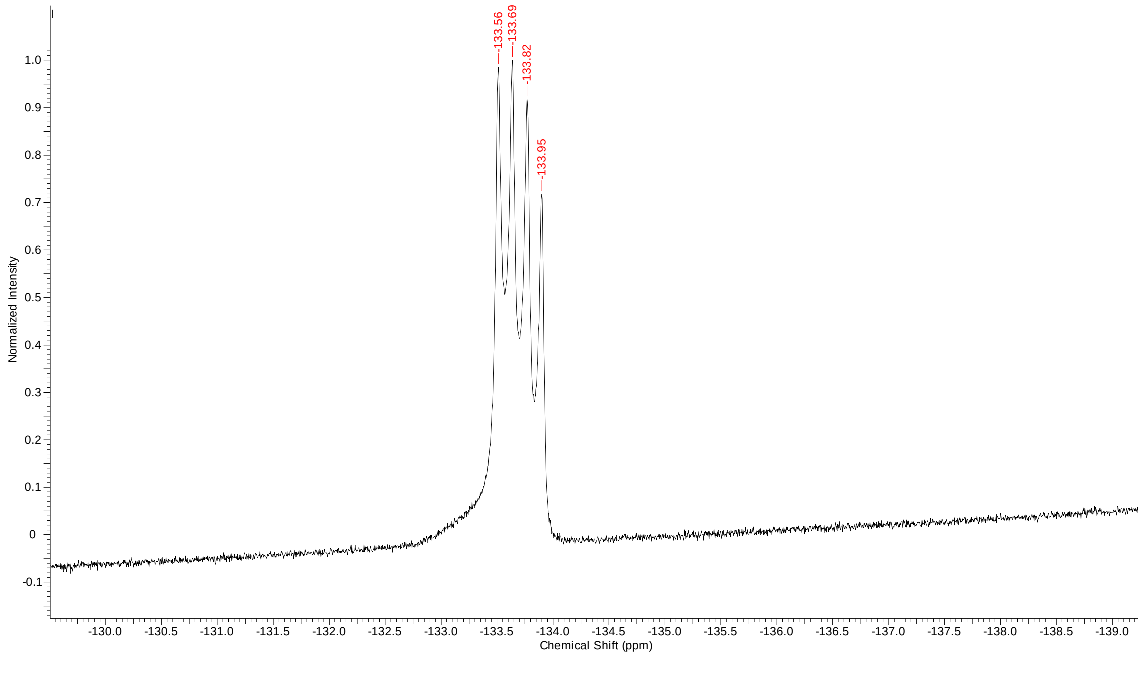

^{11}B NMR spectrum (128 MHz, DMSO-d_6) of compound **4**
